# Discrimination of Hover Fly Species and Sexes by Wing Interference Signals

**DOI:** 10.1002/advs.202304657

**Published:** 2023-10-17

**Authors:** Meng Li, Anna Runemark, Julio Hernandez, Jadranka Rota, Rune Bygebjerg, Mikkel Brydegaard

**Affiliations:** ^1^ Department of Physics Lund University Sölvegatan 14c Lund 22363 Sweden; ^2^ Department of Biology Lund University Sölvegatan 35 Lund 22362 Sweden; ^3^ Norsk Elektro Optikk Østensjøveien 34 Oslo 0667 Norway; ^4^ Biological Museum, Department of Biology Lund University Sölvegatan 37 Lund 22362 Sweden; ^5^ FaunaPhotonics Støberigade 14 Copenhagen 2450 Denmark

**Keywords:** biodiversity, hover fly, hyperspectral, insect wing, wing interference signals (WISs)

## Abstract

Remote automated surveillance of insect abundance and diversity is poised to revolutionize insect decline studies. The study reveals spectral analysis of thin‐film wing interference signals (WISs) can discriminate free‐flying insects beyond what can be accomplished by machine vision. Detectable by photonic sensors, WISs are robust indicators enabling species and sex identification. The first quantitative survey of insect wing thickness and modulation through shortwave‐infrared hyperspectral imaging of 600 wings from 30 hover fly species is presented. Fringy spectral reflectance of WIS can be explained by four optical parameters, including membrane thickness. Using a Naïve Bayes Classifier with five parameters that can be retrieved remotely, 91% is achieved accuracy in identification of species and sexes. WIS‐based surveillance is therefore a potent tool for remote insect identification and surveillance.

## Graphical abstract

Using infrared hyperspectral imaging, a study is pioneered on wing interference signals (WISs) from hover fly wings, distinguishing nanoscopic thicknesses across 30 species and sexes. The analysis boasts an impressive 91% identification accuracy for both species and sexes. Intriguingly, these nano‐features correlate with ecological traits, deepening the understanding of hover fly biology and promising advancements in automated pollinator surveillance.

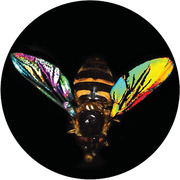

## 1 Introduction

Insect diversity and abundance have been significantly reduced by anthropogenic changes,^[^ ^1^, ^2^, ^3^ ^]^ such as habitat loss driven by agricultural intensification, pollution, and global warming, with insects experiencing significant declines even within protected areas.^[^ ^4^ ^]^ Insects have important ecological roles, crucial not only for natural ecosystems but also for agriculture.^[^ ^5^, ^6^ ^]^ For instance, pollination is necessary for the majority of the food crops produced today.^[^ ^7^ ^]^ Efficient pollinator groups include bees and bumblebees^[^ ^8^, ^9^ ^]^ (Hymenoptera), butterflies and moths^[^ ^10^, ^11^ ^]^ (Lepidoptera), and flies^[^ ^12^ ^]^ (Diptera), including hover flies^[^ ^13^, ^14^ ^]^ (Syrphidae). Different groups of pollinators may respond differently to anthropogenic change, with specialist insects typically showing higher sensitivity to habitat alterations.^[^ ^15^, ^16^ ^]^ Therefore, understanding the responses of single species and groups is important. Hover flies are considered the second most important pollinators after wild bees,^[^ ^17^ ^]^ and hover fly pollination is thought to be relatively resistant to fluctuating environmental conditions.^[^ ^13^ ^]^ To understand the impact of diversity and abundance of pollinators in ecosystems, it is necessary to efficiently assess all important pollinator groups, including hover flies. However, monitoring insect abundance and diversity remains a challenge for researchers.

Conventional methods for surveillance of insect diversity and abundance are time‐consuming and do not efficiently monitor all free‐flying insects. Malaise traps are considered to capture the least biased species composition of flying insects^[^ ^18^ ^]^ and pan‐traps are used to monitor the Hymenoptera and Diptera pollinators.^[^ ^19^ ^]^ These methods are labor‐intensive and require taxonomic expertise for insect identification.^[^ ^20^, ^21^ ^]^ Environmental DNA (eDNA) monitoring^[^ ^22^, ^23^, ^24^ ^]^ does not provide abundance estimates or insights into activity patterns. Machine vision approaches can provide species‐level identification, but only of caught insects^[^ ^24^ ^]^ and pollinating visitors in the field,^[^ ^8^, ^25^ ^]^ as free‐flying insects cause focus and motion blur issues. Thus, current image recognition approaches rely on trap designs and baits, and they yield a low number of observations to resolve weather and hourly niches.

To improve species identification of free‐flying insects, the frequency‐, polarimetric‐ and spectral domains have been explored,^[^ ^26^ ^]^ and real‐time field sensors that discriminate free‐flying insect species based on wing beat frequencies (WBFs) and harmonic overtones developed.^[^ ^27^, ^28^, ^29^, ^30^ ^]^ WBF and harmonic spectrum depend on both the wing dynamics,^[^ ^31^ ^]^ wing surface roughness,^[^ ^11^ ^]^ and wing membrane thickness^[^ ^32^, ^33^, ^34^ ^]^ in relation to the wavelength. The WBFs of hover flies are in the 150–300 Hz range,^[^ ^35^ ^]^ which is between the WBFs of bees^[^ ^36^ ^]^ and mosquitoes.^[^ ^35^ ^]^ However, the WBF depend on environment temperature,^[^ ^29^, ^36^, ^37^ ^]^ humidity,^[^ ^36^ ^]^ and body mass.^[^ ^9^, ^38^ ^]^ Even for a constant temperature, the relative spread of the WBF^[^ ^37^, ^39^ ^]^ for a single species and sex is typically 25%, in the best case this would leave room to distinguish 3–4 species or sex of hover flies (log(300 Hz/150 Hz)/25%). Therefore, utilizing WBF alone does not enable differentiation among the hundreds of coexisting species of syrphid flies.

In order to improve the discrimination of free‐flying species of insects, photonic modulation methodology can be expanded in dimensionality by adding polarimetric‐ and spectral bands. The polarimetric domain provides sensitivity to microstructural features,^[^ ^34^, ^40^ ^]^ whereas different spectral bands^[^ ^41^ ^]^ may yield molecular or nanoscopic information on the wing membrane thickness.^[^ ^32^, ^33^ ^]^ Importantly, most of the light backscattered by insects is oscillatory and contributed by insect wings,^[^ ^39^, ^42^ ^]^ and most of that light is scattered by specular reflections in insect wings. Thus, specular flashes can be observed by insects in flight.^[^ ^34^ ^]^ Spectrally, this wing flash is dominated by fringy thin‐film interference resonances^[^ ^32^, ^34^ ^]^ referred to as a wing interference signal (WIS) in this study. The flash instances represent the rare occasions when the wing orientation is entirely known and the reflectance spectra^[^ ^34^, ^43^ ^]^ may provide the quantitative information to determine the species and sex. Existing literature on wing interference patterns (WIPs) includes comparative studies^,[^ ^44^, ^45^ ^]^ with RGB color cameras or spectroscopic case studies for single insect specimens.^[^ ^34^, ^46^ ^]^ WIPs are reported as species‐specific, sexually dimorphic in blow flies,^[^ ^47^ ^]^ and stable over time.^[^ ^44^ ^]^ However, no literature provides quantitative estimates of the thicknesses, statistics across the entire wings, or any clues on how the differences between species compare to the within‐species variation. We previously reported values for membrane thickness for various families,^[^ ^33^ ^]^ but that study did not include replicates within species, and the significance of species contrast could therefore not be evaluated.

Here, we address how the nanoscopic features extracted from WIS can improve the species and sex identification of hover flies. We used infrared hyperspectral imaging and scanned a total of 600 wings from 30 species of pinned hover flies, including five replicates of each sex, and both the left‐right wings. To our knowledge, no studies quantitatively determined membrane thickness or its within‐species variation and how it can be used for differentiating insect species. Here, we ask to what extent the membrane thickness differs within wings and if wing thickness and variation in thickness differ among species and sexes. We assess how the membrane thickness scales with wing area and test if genetic divergence or ecological niche explains the variation in wing thickness and modulation. A challenge for interpreting signals is to document how fringe modulation depth and fringe heterogeneity vary across and within species and sexes. This determines the applicability of WIS for species identification and signaling. Based on thin‐film theory, we explain WIS by four parameters (membrane thickness, thickness heterogeneity, fringe amplitude, and ‐bias). We uncover that wings as a whole produce fringy spectra, that the thickness differs significantly among species and sexes, and ecological traits explain variation in WIS better than genetic distance. This paves the way for improved specificity and species identification of insects using photonic entomological sensors with spectral sensitivity. This can imply the applicability of insect monitoring techniques across species and could greatly improve our understanding of the factors affecting insect diversity and abundance.

## 2 Results and Discussion

### 2.1 Wing Interference Fringes Survive Spatial Averaging over the Wing Surfaces

**Figure 1:**
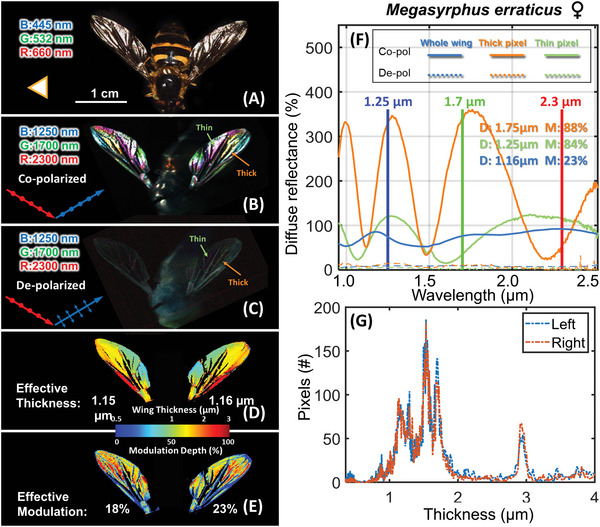
RGB and false color images of the hover fly *M. erraticus*. A) A photo of female *M.erraticus*. B,C) Two false color images were generated for co‐ and depolarizations with false color bands chosen according to the hyperspectral data cube (band choices: blue @ 1250 nm, green @ 1700 nm, and red @ 2300 nm) with a gain of 80%. D,E) Membrane thickness and modulation depth maps, showing the reflectance of the selected thick and thin wing pixels in (B,C). The blue colored reflectance spectrum in (F) corresponds to the effective wing reflectance integrated over the whole left wing. The orange and green reflectance spectra corresponding to the fringes form the thick and thin wing pixels. *D*: thickness, *MD*: modulation depth. G) Histograms of the wing thickness distributions of all left‐ and right‐wing pixels.

A polarimetric shortwave infrared hyperspectral camera (0.95<*λ*<2.5 µm, details see Section 4.4) was used to capture the WIS from the entire wings of the mounted individuals. Each pinned individual was scanned by a polarimetric shortwave infrared hyperspectral imager by specular illumination configurations (see example in **Figure** **1**). The visual appearance of the broad spectral bands of the ordinary RGB color camera (Figure 1A) fails to resolve the narrow interference fringes spectrally, and consequently, the wings appear whitish. In contrast, a false color image based on selected narrow infrared bands results in soap bubble colors over the wing surface (Figure 1B). These WIPs are highly coherent, directional, and thus co‐polarized as illustrated by the negligible de‐polarized light (Figure 1C). Spectral fringes, caused by thin film interference,^[^ ^33^, ^46^, ^48^ ^]^ differ between thick and thin wing sections (Figure 1F). The WIPs (as shown in Figure 1B) are reflected from the chitin membrane, whose structure remains unchanged from living adult flies to pinned museum specimens.^[^ ^44^ ^]^ As the specular WIS is highly directional, the resonant reflectances exceed the white Lambertian reference (100% diffuse reflectance). This implies that WIS could be detectable over extended distances with a considerable contrast against background,^[^ ^32^, ^34^ ^]^ with consequences both for visual ecology and the prospects for detection and monitoring. The spectra obtained from an individual pixel exhibit significant fluctuations, reaching up to 88% modulation (Figure 1F, orange solid line, see also Section 4.5). In contrast, the de‐polarized contribution shows minimal reflectance of a few percent, (See Figure 1F orange dash line). By averaging the reflectance across the entire wing, the spectral modulation decreases to 23% (Figure 1F, blue solid line). We conclude that the reduced spectral modulation for whole wings is due to the dephasing of fringes from distinct membrane thicknesses across the wing surface. As a result, thinner wings and longer wavelengths generally exhibit higher levels of spectral modulation. However, fringy properties are still detected in all species examined, enabling the estimation of effective thickness for the whole wings (Figure 1D). Generally, clear insect wings are thick toward the anterior edge and thin toward the posterior edge. The left‐right discrepancy of the effective thickness is just 10 nm, values are shown in Figure 1D for the examined individual, similar to the precisions achieved by lidar estimates on free‐flying insects.^[^ ^32^ ^]^ The spectral modulation depth varies across the wing surface (Figure 1E), implying that all parts of the wing surfaces does not contribute equally to the effective fringe. In particular, the wing veins do not produce spectral fringes and display low modulation, as illustrated by the histogram of wing thicknesses in individual pixels (Figure 1G). The histograms from the right and left wings are highly consistent. Finally, the effective thickness of 1.15 µm is somewhat thinner than the most common thicknesses encountered on the same wing. This is because the thinner areas of the wing have higher modulation values and fringes from thinner membranes are more prone to interfere constructively.

### 2.2 Wing Interference Signals are Species‐ and Sex‐Specific

**Figure 2:**
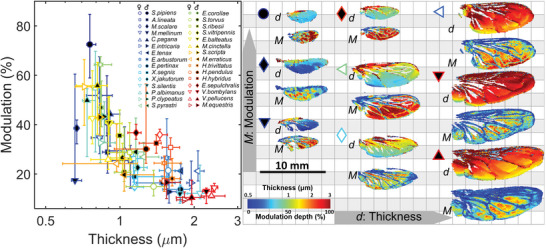
Membrane thickness and fringe modulations scatter plot. A) Effective membrane thickness versus spectral fringe modulation depth. The markers indicate median values and the bars indicate the ¼ and ¾ quartiles of the within‐species and sex variation. The full name of the species in the legend is listed in Table S1 (Supporting Information). The species color coding is sorted by minimizing genetic distance with their neighbors in Figure S1 (Supporting Information). B) Nine representative cases showing the membrane thickness and modulation depth maps.

Wings from five individuals of each sex from 30 hover fly species were analyzed. The wing surfaces were spatially averaged, and effective fringes were obtained (Figure 1). The effective fringes or WIS (**Figure** **2**A) can be explained by a spectral model with four degrees of freedom; an effective thickness, *d*_wing_, the thickness heterogeneity across the wing surface, *λ*_0_, a fringe amplitude, *α*, and a bias term, *β*. For convenience, we also measure the spectral modulation depth, *M*, within the spectral window of our instrument. Determining the effective thicknesses and modulations for each sex and species, we find that thickness ranged between 0.4 and 2.2 µm (Figure 2A; Figures S1 and S2, Supporting Information), and effective modulation depth in the short‐wave infrared varied from 10 to 70%. Thicker wings are not highly modulated, whereas some thin‐winged species display a variety of modulation depths.

In contrast to the frequency domain,^[^ ^35^ ^]^ we found that among species differences were more pronounced than differences within species and between sexes. While many species overlap with their neighbors in this 2D scatterplot, most species do not have any overlap with the majority of the other 29 examined species. Species‐ and sex differences were apparent in both the effective membrane thickness, *d*_wing_, and effective fringe modulation depth, *M* (Figure 2A). We also observed differences in the other WIS properties such as effective fringe heterogeneity, *λ*_0_, effective fringe amplitude, *α*, and bias *β* (see Figures S3–S5, Supporting Information). The differences have interesting implications, primarily for the feasibility of using spectral technology to differentiate free‐flying species but also for signaling in visual ecology. As indicated by the resemblance of species with similar color codes, more closely related species across tribes and genera have more similar WIS properties. However, there is no obvious pattern of thickness across the entire hover fly family. To illustrate the substantial variation in membrane thickness and modulation across species (Figure 2A), we selected nine representative cases and presented their membrane thickness maps and corresponding modulation maps (Figure 2B). Generally, insect wings are thinner toward the rear edge, which also primarily contributes to the effective fringe and modulation. We found this pattern to hold true across both species and sexes, but different species and sexes displayed different degrees of gradient changes in thickness and modulation variation (Figure 2B). Finally, our results showed that thin‐winged species are more likely to produce WISs with strong modulation depths (Figure 2A; Figure S6, Supporting Information).

**Figure 3:**
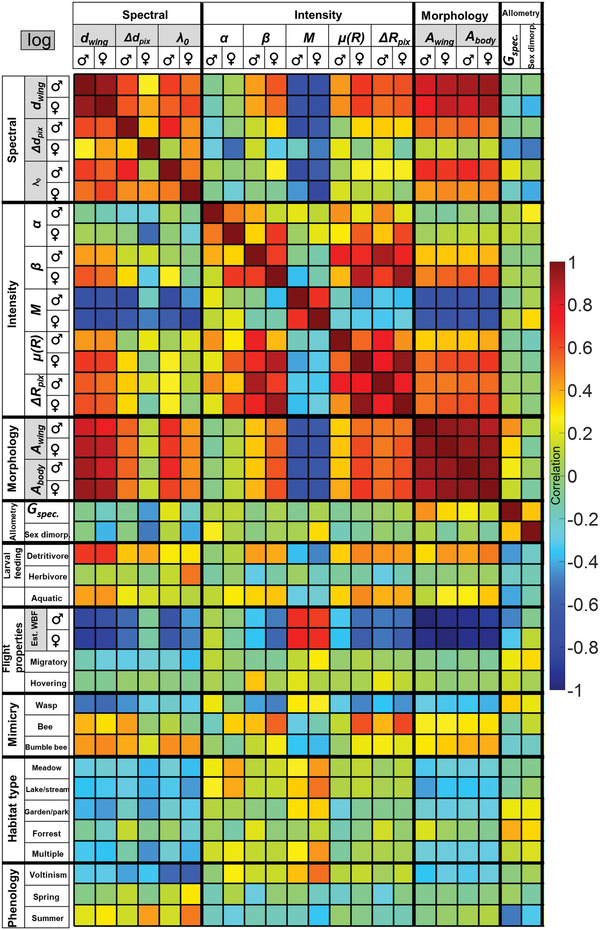
A correlation coefficient matrix of WIS parameters with ecological traits (cropped version, full matrix is shown in Figure S7, Supporting Information). The maximum positive correlation is 1, the maximum negative correlation is −1 and the minimum correlation is 0. Definition of abbreviations; *d*_wing:_ membrane thickness_,_ *Δd*_pix:_ wing heterogeneity, *λ*_0_: fringe heterogeneity, *α*:effective fringe amplitude, *β*: effective fringe bias, *M*: fringe modulation depth, *µ(R)*: broadband reflectance, *ΔR*_pix_: degree of wrinkling, *A*_wing_: wing area, *A*_body_: body area, *γ*: allometric power relation, Est.WBF: estimated wing beat frequency.

One study^[^ ^49^ ^]^ suggested that subwavelength gradient refractive indices on wing surfaces reduce modulation depths of fringes and WISs, and others have suggested that wrinkled wings distribute interference signals spherically.^[^ ^44^ ^]^ To uncover the main limiting factor for the effective modulation of hover fly wing fringes, we introduced the mean broadband reflectance, *µ(R*_λ_ *)*, for the wings and calculated the broadband reflectance spatial standard deviation reflectance, *ΔR*_pix_, as a measure of wrinkles. We correlated the modulation depth *M* with the wing heterogeneity, *Δd*_pix_, fringe heterogeneity, *λ*_0_, broadband reflectance, *µ(R*_λ_), and degree of wrinkling, *ΔR*_pix_. We employed the Pearson correlation coefficient, and positive definite quantities were logarithmized prior to correlation (marked in gray in **Figure** **3**). Our study incorporated all 30 species, and distinctions were made solely when correlating males and females individually, as represented by the respective sex symbols in the figure. We found that modulation was primarily related to membrane thickness heterogeneity *Δd*_pix_ and *λ*_0_, and less to *µ(R*_λ_) and *ΔR*_pix_ (Figure 3). Modulation damping by thickness heterogeneity takes effect when widely distinct membrane thicknesses across the surface are averaged and interfere destructively. This effect predominantly damps the WISs toward shorter wavelengths because thin‐film WISs are chirped, and the higher‐order modes toward shorter wavelengths are more sensitive to dephasing. Thus, we concluded that the effective fringe modulation depth is primarily influenced by the heterogeneity in wing thickness compared to other nano features such as wrinkles and anti‐reflective gradient refractive indices.

### 2.3 The Allometric Relationship between Wing Area and Membrane Thickness is Not Isometric

**Figure 4:**
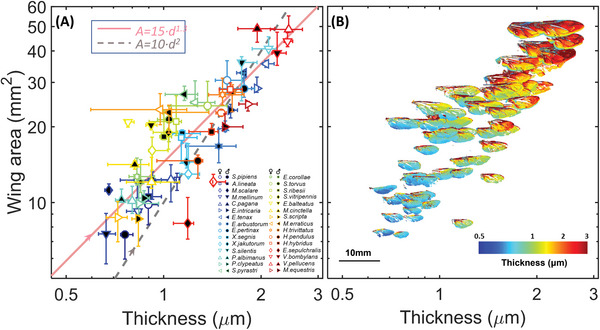
Wing area and membrane thickness are not isometrically related. A) Effective membrane thickness versus wing area. The power relation *A*_wing_ *= 15∙d*_wing_ *^1.3^* represents the allometric relationship between the wing area and thickness better than *A*_wing_ *= 10∙d*_wing_ *^2^*. B) Wing thickness map for each species and sex based on their medians in (A). The scale bar is for the scaling of wing thickness maps.

Projected wing‐ and body‐area are available for both machine vision, wingbeat sensors, and lidars. To assess whether the membrane thickness provides complementary information or scales with wing area, we investigated the relationship in the examined hover flies (**Figure** **4**A,B). We found the relationship to be described by a power relation of the form *A*_wing_ *= G*_all_ *d*_wing_ *^γ^*, where *γ* = 1.3 [1.2–1.4] and the *aspect ratio G*_all_ = 15 (14–16, 95% confidence interval, and R^2^_adj_ of 80%; Figure 4A). This relationship is significantly different from isometric scaling with the form *A*_wing_ *= G d*_wing_ *^2^*, where *G* = 10 (9–11, 95% confidence, and R^2^_adj_ of only 56%; Figure 4A). Despite the strong covariance between wing area and thickness, many species deviate from the allometric expectation providing additional species‐specific signals. Notably, some larger flying insects, such as dragonflies,^[^ ^34^ ^]^ have wing areas of 700 mm^2^ but similar wing thicknesses of 2–3 µm. Another study found submicron thicknesses of equally large grasshopper wings.^[^ ^32^ ^]^ These examples illustrate how thickness‐to‐area aspect ratio and allometric relation can be entirely different for other orders and families.^[^ ^33^ ^]^ Our study highlights the diversity of membrane thickness maps in relation to wing sizes (Figure 4B).

Interestingly, although wing area and membrane thickness covary, the two parameters also hold complementary species‐specific information as illustrated by the correlation matrix including WIS parameters to wing‐ and body area (Figure 3). The wing membrane thickness *d*_wing_ correlates with both the wing area *A*_wing_ and body area *A*_body_ (Figure 3). Furthermore, the wing membrane thickness is anti‐correlated with the modulation depth, *M*, and estimated WBF, *f̂*, (Figure 3; Figure S7, Supporting Information). The WBF, *f̂*, was estimated based on hover flies body mass and wing area (details are reported in the Experimental Section). Overall, the correlations between WIS parameters, wing, and body area suggest hover flies with large bodies are more prone to have larger and thicker wings, a lower WBF, and weak WIS modulation.

Our results demonstrate that the membrane thickness, heterogeneity, and modulation provide complementary information on wing area. The wing and body area can be retrieved from free‐flying insects with existing photonic sensors^[^ ^50^ ^]^ and lidar technique^[^ ^51^ ^]^ (as projected scattering cross‐section in mm^2^). Therefore, it is feasible to expand the capability of lidar to capture the membrane thickness of in‐flight insects and use it as complementary information to significantly improve species identification. Although this study reports measurements from mounted individuals with known illumination geometry, the results can be translated to field observations. This is because backscattered signals from clear‐winged insects are dominated by the specular WIS flash^[^ ^34^ ^]^ and because the wing surface orientation is known in the instance a WIS flash occurs. In a recent report,^[^ ^32^ ^]^ we demonstrated that such signals can be retrieved remotely in the field, that the membrane thickness can be estimated, and that this thickness is consistent with laboratory recordings. This implies that any photonic device measuring backscattering from insects would favor the detection of species with resonant wings for the employed wavelength bands.

### 2.4 Classification Accuracies Based on Interference Signals

We concluded that the light reflected by insect wings is sensitive to the membrane thickness and that this thickness differs greatly between species. Moreover, most hover fly species exhibit sexually dimorphic WIS properties (Figures 3 and 4; Figures S3–S5, Supporting Information). Interestingly, this also holds true for species where sex determination based on morphology and size is known to be challenging (Table S1, Supporting Information).

We evaluated the benefit of including spectral information for species‐ and sex‐specificity of photonic‐based sensors. We combined thickness *d*_wing_ and *λ*_0_ with several features, including the WBF, *f̂*, wing area *A*_wing,_ and modulation depth *M* (see methods*)*. We included *f̂* and *A*_wing_ because they are common features for insect identification with photonic sensors,^[^ ^52^, ^53^, ^54^, ^55^ ^]^ the spectral parameters *d*_wing_ and *λ*_0_ can potentially be determined with great accuracy,^[^ ^32^ ^]^ and the modulation, *M*, is a unitless feature that can be extracted from a WIS. In addition, a WIS with a low *M* implies great uncertainty of the fringe parameters. We applied a Naïve Bayesian Classifier (NBC), a classification method simply estimating the overlap of groups in an N‐dimensional parameter space on these parameters, specified in metric units. We show that the successful identification rate based on the WBF, *f̂*, alone was only 13%, but 91% when all four features were considered (**Table** **1**). We note that *λ*_0_ and *M* could not be estimated without simultaneously estimating *d*_wing_ This improvement demonstrates that the deduced spectral information, such as the membrane thickness *d*_wing_ and modulation depth *M*, is complementary and could revolutionize remote species identification using WISs. Several studies have explored the discrimination of free‐flying insects by their oscillatory properties using a single band,^[^ ^54^, ^56^, ^57^, ^58^, ^59^ ^]^ and our findings show that specificity can be strongly improved by adding up to four spectral bands according to the fringe model. For single‐band instruments, our findings imply increased signal‐noise‐ration or detection range for species with resonant wings for the particular wavelength. The resonant condition is given by *λ*_max_ = 2*nd*_wing_/(*m*‐½), *m*∈ℕ, as an example, the most brilliant of our hoverflies *Syritta pipiens* has *d*_wing_ of 762 nm and resonant backscatter at 670, 925, and 1546 nm wavelength. The half mode term in the equation derives from the 180˚ phase shift from the first air‐chitin reflection (this flip is absent from the backside chitin‐air reflection).

**Table 1:** Accuracy of identifying the correct species and sex of hover flies based on one or several chosen parameters, more details see Figures S12–S20 (Supporting Information). WBF: wing beat frequency, *d*_wing_: effective membrane thickness, *M*: fringe modulation depth, *λ*_0_: fringe heterogeneity*, A*_wing_: wing area.

Accuracy [%]	*WBF* [Hz]	*d*_wing_ [µm]	*λ*_0_ [µm]	*A*_wing_ [mm^2^]	*M* [%]
13	✓				
22		✓			
35		✓			✓
40		✓	✓		
63	✓	✓			
74	✓	✓	✓		
75	✓	✓			✓
85	✓	✓		✓	✓
91	✓	✓	✓	✓	✓

### 2.5 Nanoscopic Features, Genetic Distance, Species Ecology, and Morphology

Using correlation analyses, we found that genetic distance between species only explained 29% and 26% of the variation in wing thickness, *d*_wing_, for males and females respectively. The genetic distance explained 31% of the variation in spectral modulation, *M*, for males, but just 8% for females. For comparison, the group of detritivorous larvae explained 35% and 37% of the difference in wing thickness for males and females respectively. Grouping the species into wasp‐ and bumblebee mimicking explained equally much of wing thickness variation as genetic relatedness, with correlation coefficients between 22 and 29% (Figures S9 and S10, Supporting Information). We also tested if differences in wing thickness‐area aspect ratios, *G*_spec._, and differences in sexual dimorphism in different species were significantly correlated with genetic distance, but found that this was not the case. There are two potential caveats to our approach. One is that we used a short fragment of the mitochondrial DNA (the DNA barcode) for estimating genetic distance, and the other is that we have only sampled a small percentage of the known hover fly species. To test a more complicated relationship between thickness and genetic relatedness, we plotted wing thickness against cumulative genetic distance from the two extreme species *S. pipiens* to *Merodon equestris* (Figure S11, Supporting Information). The analysis revealed that the wing thickness parameter folds twice across the hover fly family (a third‐order polynomial yielded 36% and 41% R^2^_adj_. for males and females, respectively). This confirms our observation that thicknesses are similar on the genus level but not on the family level (Figure 2A).

In addition, we tested whether ecological niche, phenology, or morphological differences influenced the nanoscopic properties or biomechanical structure of hover fly wings, which would increase the ability to interpret signals from unknown taxa in field studies. Hover flies are often generalist or broad‐spectrum flower visitors,^[^ ^60^, ^61^ ^]^ but differ in phenology, habitat use, larval diet,^[^ ^62^ ^]^ and morphology. For instance, some hover fly species mimic bumble bees, whereas others mimic wasps, with considerably distinct wings and WISs. The larval diets of Syrphidae span carnivore aphid predators, aquatic detritus feeders, and herbivores. Moreover, hover fly sexes exhibit distinct behaviors, with females collecting more pollen‐containing proteins for egg production and males establishing territories and fiercely defending them from intruders^[^ ^63^ ^]^ through hoovering. To understand how the species morphology and ecology predict WIS parameters, we investigated the relationship between larval feeding habits, larval habitats, flight properties, mimicry, habitat type, and phenology of the focal species to our four WIS parameters (Figure 3; the full matrix is shown in Figure S7, Supporting Information). We found that hover flies with thicker wings are: a) more likely to be mimics of bees and bumble bees (rather than wasps), b) less likely to be found near lakes/streams or gardens/parks, c) more likely to have detritivorous larvae, and d) tend to be more active during summer than during other seasons. Since thinner wings are more strongly modulated, we generally encountered anticorrelations between these ecological traits and spectral modulation, *M*. Hover flies with higher spectral modulation, are a) more likely to mimic wasps, b) prefer diverse habitats, including meadows, c) are more likely to have terrestrial and carnivorous larvae, and d) have more generations within a year and less active during summer. These findings can potentially provide clues about the traits of the hover fly species observed based on WIS observations. For instance, if larval feeding habits are reflected by membrane thickness, it could have applications for sensors in agriculture.

Finally, we addressed if species deviated from the general allometric relation by estimating a species‐specific wing thickness‐area aspect ratio, *G*_spec._ = *Ad^−γ^* (Figure 4; Figure S8, Supporting Information). We found that large‐winged species with thinner wings than expected by the allometric relationship have a tendency to be more sexually dimorphic (36% of the variation is explained by this correlation), and males generally have larger wings than females with sex explaining 46% of the within‐species variation in data (Figure 4A). This could potentially be due to selection for sexually selected visual signaling, as proposed in earlier work,^[^ ^45^ ^]^ or reflect sex‐specific behaviors or morphology. Hover flies with large thin wings prefer forest habitats (40%), whereas hover flies with thick small wings are more likely to have detritivorous (43%) or aquatic larvae (41%), and be active during the summer month (50%; Figure 3 row *G*_spec_; see also Figures S7–S8, Supporting Information).

## 3 Conclusion

We presented a new and innovative approach that could revolutionize our ability to remotely identify species and sex of flying hover flies. We can explain wing interference signals by four parameters, including membrane thickness and thickness heterogeneity. Using these parameters, we successfully distinguished 30 different species of hover flies and could discriminate between sexes. The proposed scheme achieves an identification accuracy of up to 91% on mounted hover flies. Similar signals can be captured from free‐flying insects in the field,^[^ ^32^ ^]^ and hence discrimination can be greatly improved by additional spectral bands in photonic insect sensors. The implications of this work are that 1) single‐band photonic sensors^[^ ^29^ ^]^ or lidars^[^ ^28^ ^]^ have preferences for species with resonant wings for the employed wavelength, 2) dual‐band systems^[^ ^30^, ^41^, ^43^ ^]^ could differentiate species according to wing thicknesses although thickness would be ambiguous, 3) unique determination of wing thickness would require minimum four spectral bands, however hyperspectral monitoring^[^ ^32^ ^]^ of insect could prove excessive and benefit from speed rather than spectral bands.

Wing features were correlated with hover fly morphology and ecology, including habitat use and larval ecology, which potentially could help classify signals from field observation from hover fly species not included in the study in the future. Our novel method for identifying hover flies is expected to make a significant contribution to the development of remote monitoring sensors for flying insects. The ability to accurately identify species and sexes of hover flies will greatly enhance our understanding of their abundance and distribution as well as habitat‐specific diversity, which ultimately can inform the development of more effective strategies for protecting these important pollinators and their habitats.

## 4 Experimental Section

### Hover Fly Specimens

A total of 300 individual hover flies from 30 hover fly species (five replicates of each sex) were provided by the Lund University Biological Museum, and both the right and left wings of each specimen were studied. While hover flies (Syrphidae) are challenging for amateur entomologists to identify, the flat and clear wings of hover flies are ideal targets for identification over long distances using photonic sensors based on spectral WISs. The 30 hover fly species were chosen based on their abundance in Sweden and wing size: species with wingspans between 15 and 40 mm were chosen to fit within the field of view of the instrument. The 30 hover fly species included in this study are listed in Table S1 (Supporting Information). The males and females of some hover fly species (e.g., *Episyrphus balteatus*), are similar in size and form.^[^ ^20^, ^21^ ^]^ These are marked gray in Table S1 (Supporting Information).

### Ecological Traits

The 30 species studied here were categorized according to the following traits covering different aspects of the hover fly biology: larval feeding habits (herbivores, carnivores, or detritivores), larval habitats (terrestrial or aquatic), flight properties (WBF, migratory and hovering), mimicry (wasp, bee, or bumblebee), habitat type (meadow, lake/stream, garden/park, forest or multiple), and phenology (voltinism, spring, or summer). Information about the ecological traits was provided by Rune Bygebjerg from the Lund University Biological Museum (see Supplementary Spreadsheet, *Hover fly listRB2.xlsx*).

### Genetic Pairwise Distance

The genetic distance between the 30 species of Syrphidae was calculated using the publicly available sequences and tools in The Barcode of Life Data System tools.^[^ ^64^ ^]^ The K2P distance model was applied on the sequences that were aligned using the BOLD Aligner. The DNA barcodes for *Anasimyia lineata* and *Cheilosia pagana* could not be found in the BOLD databases; thus, they were replaced by proxy congener species (*Anasimyia lunulata* and *Cheilosia albipila*) to complete the pairwise distance comparisons. The result of these pairwise genetic distances is visualized in Figure S1 (Supporting Information), where the species are sorted by maximizing neighbor similarity. It is noted that the use of a proxy species could weaken the strength of the genetic results.

### Capturing Spectral WISs with a Hyperspectral Camera

A polarimetric Short‐wave infrared (SWIR) hyperspectral imaging system (HySpex: SWIR‐384, Norsk Elektro Optikk AS, Norway) similar to that used in previous studies^[^ ^11^, ^33^, ^65^ ^]^ was used to image the hover fly wings. The hyperspectral camera covered the shortwave infrared wavelength range from 0.95 to 2.5 µm and a spatial resolution of 240 µm/pix, with 288 spectral bands. A broadband halogen‐tungsten lamp (150 W) placed 30 cm away from the sample was used for illumination (impinging on the pixel footprints in a ±8° light cone). The camera and light source were arranged in a specular condition with ±56° (Brewster angle of chitin) to the hover fly wing surface. This angle was taken into account when calculating membrane thickness, but similar fringes can be measured at any backscatter angle. In fact, smaller incidence angles as in lidar^[^ ^32^ ^]^ ease the constraints for flat wings. Two ultra‐broadband polarizers^[^ ^66^ ^]^ were used to capture the co‐ and de‐polarized reflectance. The objective of the camera had an aperture of Ø20 mm and a working distance of 8 cm (numerical aperture of ±7° imaging cone). The swath width was 40 mm for the imaged specimen. All hyperspectral images were calibrated to a Lambertian gray standard reference (Spectralon®) of 50% reflectance. Calibrating the shiny wings to a diffused Lambertian standard implies that the specular reflectance of the wings can exceed 100% diffuse reflectance. All hover flies had their wings spread and were mounted on black neoprene, resulting in a horizontal wing surface. Black neoprene was used to reduce the amount of background light.

### Parametrizing Spectral Fringes with the Modulation and Fringe Model

The reflectance of clear insect wings can be explained by thin film interference.^[^ ^46^ ^]^ According to this, light may resonate in backscatter or transmission depending on the wavelength, membrane thickness, refractive index, and incident angle. For the case of coaxial lidar, resonant backscatter is achieved at the wavelengths *λ*_Rmax_ = 2*nd*/(*m*‐½) where as no resonance is observed at *λ*_Rmin_ = 2*nd*/*m*, where *n* is the refractive index, *d* membrane thickness and *m*∈ℕ. For all wavelengths, each wing pixel in the hyperspectral images had a corresponding spectral profile referred to as a fringe; thick and thin wing pixel examples are shown in Figure 1F, and their corresponding spectral fringes are shown in Figure 1F. The effective fringe was acquired by spatially integrating all wing pixel spectral profiles. All three example fringes shown in Figure 1F show different degrees of modulation, which can be described by the modulation depth *M* with the following equation:

(1) $$M\;=\frac{\sigma_{\lambda }\left(R_{\lambda }\right)\cdot \mu_{\lambda }\left(F_{\lambda }\right)}{\sigma_{\lambda }\left(F_{\lambda }\right)\cdot \mu_{\lambda }\left(R_{\lambda }\right)}\;$$

where *R* denotes measured reflectance, *F* denotes the computed fringe, *λ* is the wavelength, *σ*_λ_ denotes standard deviation in the spectral domain, and *µ*_λ_ is the spectral mean value. The computed fringe *F*(*λ, d*_pix_) can be calculated^[^ ^48^ ^]^ according to the membrane thickness *d*_pix_ as follows:

(2) $$F\left(\lambda ,d_{pix}\right)\;=\frac{4R_{s}\sin^{2}\left(2\pi d_{pix}\sqrt{n^{2}-\sin^{2}\theta }/\lambda \right)}{{\left(1-R_{s}\right)}^{2}+4R_{s}\sin^{2}\left(2\pi d_{pix}\sqrt{n^{2}-\sin^{2}\theta }/\lambda \right)}\;$$

where *n* is the refractive index of chitin, *θ* is the light incident angle on the membrane, which was 56° in the recordings, and *R*_s_ is the reflectance coefficient, which can be determined with the Fresnel equations. Only the S‐polarization reflectance *R*_s_ was included in the fringe model, as the P‐polarization reflectance *R*_p_ was absent when measuring specular light at the Brewster angle. The reflection coefficient *R*_s_ is expressed as:

(3) $$R_{s}={\left(\frac{\cos\theta -\sqrt{n^{2}-\sin^{2}\theta }}{\cos\theta +\sqrt{n^{2}-\sin^{2}\theta }}\right)}^{2}\;$$

The refractive index of chitin *n* at a given wavelength *λ* can be calculated^[^ ^46^ ^]^ as:

(4) $$n\;=k_{0}\;+k_{1}/\lambda^{2}$$

where *k*_0_ = 1.517 and *k*_1_ = 8800 nm^2^. It is noted that the refractive index is generally given by the density of the medium^[^ ^67^ ^]^ and further by spectral dispersion described by the Kramers–Kronig relation. Since the SWIR range is far from chitin's main absorption band at 280 nm, the refractive index only varies between 1.527 and 1.518 (<1% change). No differences are expected in refractive index between living‐ and preserved specimens. The modulation depth *M* was calculated for all three fringe examples shown in Figure 1F, and the values are shown in the same figure. The fringe model *F(λ, d*_pix_) shown in Equation (2) was used to determine the membrane thickness for fringes in all pixels. This was done by computing 1000 fringes by using the fringe model *F(λ, d*_pix_) for membrane thicknesses ranging from 0.35 to 4 µm. The thinnest observable fringes are given by the instrument's spectral range and the thickest observable fringes by the instrument's spectral resolution. For robust numerical fitting to signals with noise, the search range should be reduced to realistic values. The range was chosen to cover the predominant thicknesses of all species and sexes (See Figure S2, Supporting Information). Minor features are also seen in Figure S2 (Supporting Information), they arise from vein pixels, which are poorly described by the fringe model. However, veins have low reflectance and thus a minimal contribution to the WIS from the whole wing.

The modulation of the fringe model *F(λ, d*_pix_) is 100% across all wavelengths, but the factor is included in Equation (1) to compensate for the arbitrary number of fringes covered by the spectral range of the instrument. The measured fringes, *R(λ)*, in each pixel was then matched with the computed fringe *F(λ, d*_pix_) based on the correlation coefficient, *C*, and fitting quality parameter, *Q*:

(5) $$C\;\left(R,F\right)=\frac{\int_{0.95}^{2.5}\left(F_{\lambda ,d_{pix}}-\mu_{\lambda }\left(F_{\lambda ,d_{pix}}\right)\right)\left(R_{\lambda }-\mu_{\lambda }\left(R_{\lambda }\right)\right)}{\sqrt[{2}]{\int_{0.95}^{2.5}{\left(F_{\lambda ,d_{pix}}-\mu_{\lambda }\left(F_{\lambda ,d_{pix}}\right)\right)}^{2}\partial \lambda \int_{0.95}^{2.5}{\left(R_{\lambda }-\mu_{\lambda }\left(R_{\lambda }\right)\right)}^{2}\partial \lambda }}\;$$

(6) $$Q\left(d_{pix}\right)\;=\;C\left(R,F\right){\left(C\left(\frac{\partial R}{\partial \lambda },\frac{\partial F}{\partial \lambda }\right)\right)}^{2}$$

The derivatives of the fitting quality parameter Q were used to neglect the slopes and the squared factor void sign flipping. The membrane thicknesses of three example WISs shown in Figure 1F were calculated, and the same model was then used to calculate the membrane thickness and modulation for each specular wing pixel in Figure 1B. The membrane thicknesses and modulation depths are presented in Figure 1D,E. Histograms of the membrane thicknesses for all wing pixels from left and ring wings are shown in Figure 1G, which demonstrates the precision of the method. The fringe model *F(λ, d*_pix_) in Equation (2) was applied to parametrize the effective fringes of the whole wings and wings with a single thickness *d*_wing_. Since fringe modulation increases toward infrared wavelengths and decreases toward visible wavelengths, a longpass function was used to describe the amplitude, whereas a shortpass function was used to describe the bias:

(7) $$F_{eff}\left(\lambda ,d_{wing}\right)\;=\frac{\alpha \cdot F\left(\lambda ,d_{wing}\right){\;}_{\cdot }\lambda^{k}+\beta \cdot \lambda_{0}^{k}\;}{\left(\lambda_{0}^{k}+\;\lambda^{k}\right)}\;$$

where *k* is the slope of the long‐ and shortpass functions, and the best value to describe all effective fringes was *k* = e = 2.71. The remaining parameters are amplitude, *α*, bias, *β*, and heterogeneity, *λ*_0_. These parameters were fitted to all recordings by a numerical search algorithm (Curve fitting toolbox, MATLAB, MathWorks, USA).

Because damaged, folded, or misaligned wings cause outliers, precision weight factor was introduced based on the left–right symmetry of each individual:

(8) $$\varepsilon_{LR}=\left(1-\frac{\left|d_{left}-d_{right}\right|}{d_{left}+d_{right}}\right)\;$$

where *d*_left_ and *d*_right_ are the effective membrane thicknesses of the left and right wings of the same specimen. This weight factor, *ε*_LR_, was used when calculating the medians and interquartile ranges (IQRs) of all WIS parameters (modulation depth *M*, membrane thickness *d*_wing_, fringe amplitude *α*, WIS bias *β*, and fringe heterogeneity *λ*_0_) for each species and sex. The weighted medians and IQRs of the WIS parameters are presented in Figure 2A, Figures S3–S5 and Tables S2–S3 (Supporting Information).

More wing surface structure information can be deduced from the hyperspectral data outside the effective spectral WISs. The wrinkling measure *ΔR*_pix_ and wing heterogeneity *Δd*_pix_ of the wing surface were determined by measuring the standard deviation of the broadband reflectance *R*_pix_ or membrane thickness *d*_pix_ of all wing pixels of a given species:

(9) $$\Delta \;R_{pix}=\sigma_{pix}\;\left(\int_{0.95}^{2.5}R_{pix}\partial \lambda \right)$$

(10) $$\Delta \;d_{pix}=\sigma_{pix}\;\left(d_{pix}\right)$$

The mean reflectance *µ(R*_λ_) of the wing surface was calculated by averaging the reflectance of all wing pixels:

(11) $$\mu \;\left(R_{\lambda }\right)=\mu_{pix,\lambda }\;\left(\int_{0.95}^{2.5}R\partial \lambda \right)$$

It was noted that these three parameters could only be measured from the hyperspectral data because they required spatial information, which was not included in the effective spectral WISs. These parameters are measued although they are not applicable for remote discrimination of insects. This is done to increase the understanding of the mechanisms affecting spectral modulation. All parameters are provided in Tables S2–S3 (Supporting Information).

### Estimating the Wing Beat Frequency of the Examined Species

The relationship between wing beat frequency, body mass, and wing area has been studied extensively and provides valuable insights into insect flight dynamics.^[^ ^35^, ^38^ ^]^ However, the literature lacks information on the wingbeat frequencies of most species and sexes of the studied hover flies. Therefore, the wingbeat frequency was estimated based on the hover fly body mass and wing area. Because the specimens were dried museum specimens, the body masses of all specimens were approximated based on their body area and wing area and correlated to the Syrphidae body mass values provided in the literature.^[^ ^35^ ^]^ The predicted wing beat frequency *f̂* for Syrphidae is calculated as^[^ ^35^, ^38^ ^]^:

(12) $$\hat{f}=\;f_{0}\;\frac{\sqrt{m}}{A_{wing}}$$

where *f*_0_ is 386.9 Hz mg^−½^mm^2^, *m* is the approximated body mass and *A*_wing_ is the wing area measured according to the hyperspectral images. These values can be found in Tables S2–S3 (Supporting Information).

### The Success Rate of Identifying the Correct Hover Fly Species and Sex Based on the WISs

In addition to the spatial and frequency information, the spectral domain was considered to greatly enhance the species specificity of the photonic sensors. One hundred synthetic data points for each of the 30 species were produced for both sexes (resulted in 6000 generated data points) based on the medians and interquartile ranges (IQRs) of the membrane thickness *d*_wing_, modulation *M*, estimated WBF *f̂* and wing area (all values are shown in Tables S2–S3, Supporting Information). The 100 synthetic data points were then input into NBC without including the covariance between parameters. The classification accuracy and overlap between the species were visualized with standard confusion matrices, as shown in Figures S12–S20 (Supporting Information).

### Allometry

A power relation formula was used to describe how wing area *A*_wing_ and membrane thickness *d* are related in hover flies.

(13) $$A_{\mathrm{wing}}=G_{\mathrm{all}}d_{\mathrm{wing}}^{\gamma }$$

The constant factor *G*_all_ and the exponent *γ* in the formula were specific to examined hoverflies and describe how changes in wing area affect membrane thickness. When the wing area to wing thickness relation was isometric, γ would be expected to equal 2. However, the analysis showed that the best fit for all data points occurred when γ was 1.3 [with a range of 1.2–1.4], indicating that the scaling relationship between wing area and membrane thickness was not isometric in these hoverflies.

### Sexual Dimorphism in WISs

The sexual dimorphism was calculated as the membrane thickness of males divided by that of females for each species.

(14) $$\Delta_{sex}=\frac{d_{wing♂}}{d_{wing♀}}\;$$

## Conflict of Interest

The authors declare no conflict of interest.

## Author Contributions

M.B., A.R., J.R., and M.L. conceptualized the idea. M.B., J.H., and M.L. designed the methodology. M.B., A.R., J.R., R.B., and M.L. performed investigation. M.B. and M.L. visualized the idea. M.B. performed funding acquisition. M.B., A.R., and J.R. performed project administration. M.B., A.R., J.R., and R.B. performed supervision. M.L. and M.B. wrote the original draft. M.B., A.R., J.R., J.H., M.L., and R.B. wrote, reviewed, and edited the final manuscript.

## Supporting information

Supporting Information

## Data Availability

The data that support the findings of this study are available in the supplementary material of this article.
