# Supplementary material for: Discrimination of Hover Fly Species and Sexes by Wing Interference Signals
Source: Adv Sci (Weinh). 2023 Oct 17;10(34):2304657. doi: 10.1002/advs.202304657 (PMC10700183; doi:10.1002/advs.202304657)
Supplement: Supplementary file 1 — Supporting Information [file ADVS-10-2304657-s001.pdf]

ADVANCED SCIENCE
Open Access

Supporting Information

for *Adv. Sci.*, DOI 10.1002/advs.202304657

Discrimination of Hover Fly Species and Sexes by Wing Interference Signals

Meng Li, Anna Runemark, Julio Hernandez, Jadranka Rota, Rune Bygebjerg and Mikkel Brydegaard*

Supporting Information

Title: Discrimination of hover fly species and sexes by wing interference signals

Meng Li[1*], *Anna Runemark*[2], *Julio Hernandez*[3], *Jadranka Rota*[4], *Rune Bygebjerg*[4], *Mikkel Brydegaard*[1,2,3,5]

Corresponding author: meng.li@forbrf.lth.se

The PDF file includes:

Figs. S1 to S20
Tables S1 to S3

Supplementary Figures:

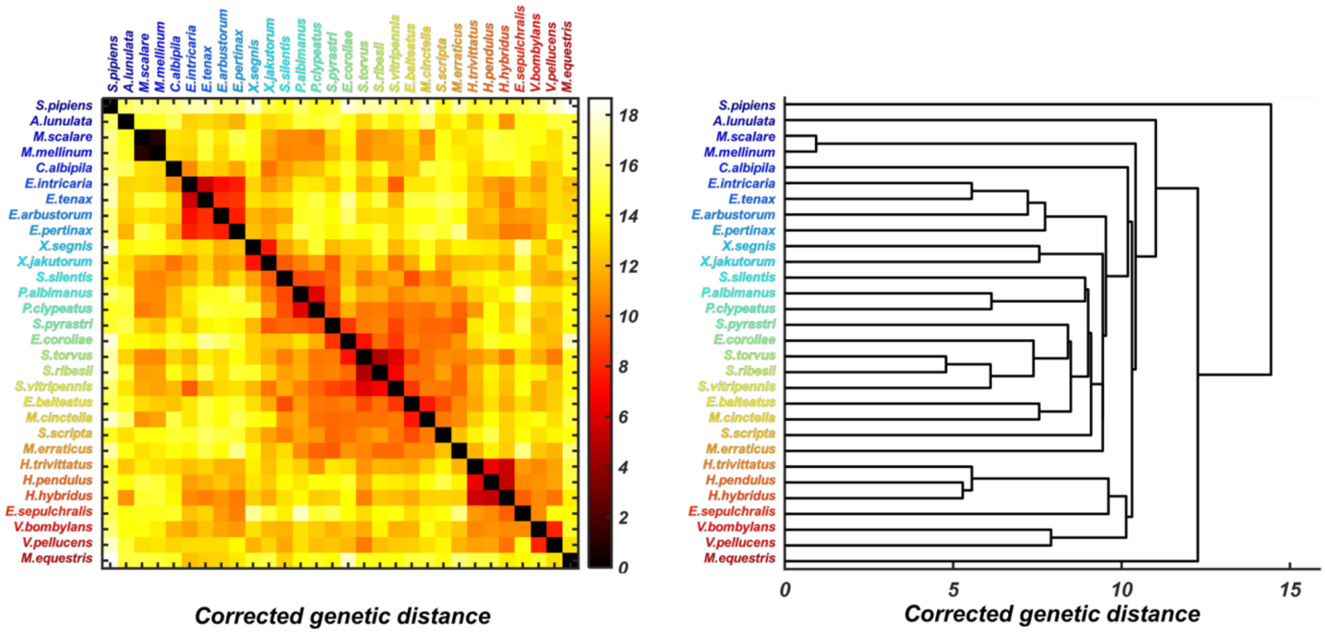

Figure S1: Corrected genetic distance between all of the pairs of the 30 studied species of hover flies is shown here with darker colours showing lower genetic distance (A); a neighbor-joining tree based on the corrected genetic distances (B). The calculated distances are given in Supplementary Spreadsheet, *pairwise_distance.xlsx*.

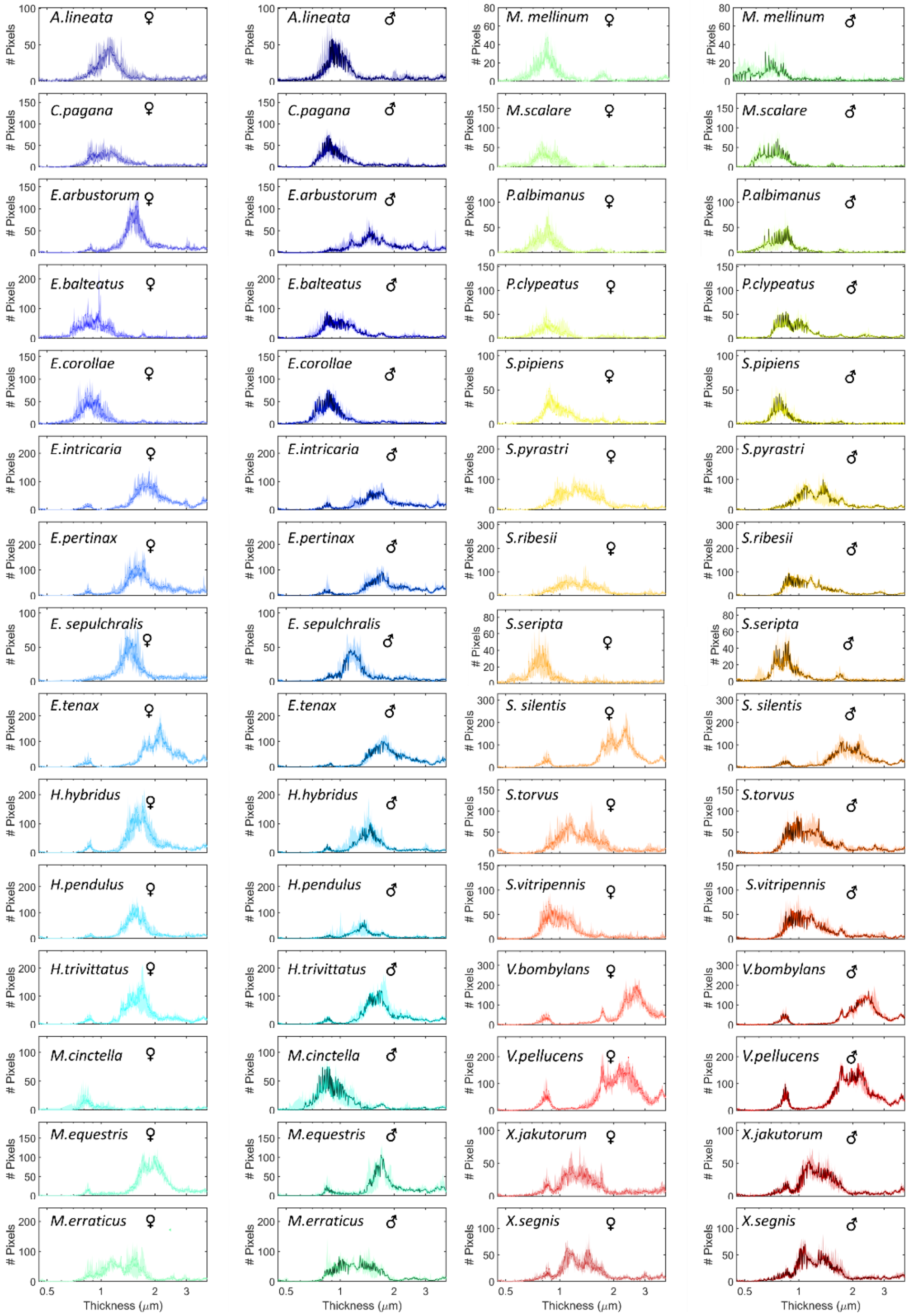

Figure S2: Membrane thickness distribution for each species and sex of 30 species of hover flies. The solid line in each plot represents the median, and the colored shading represents the variance.

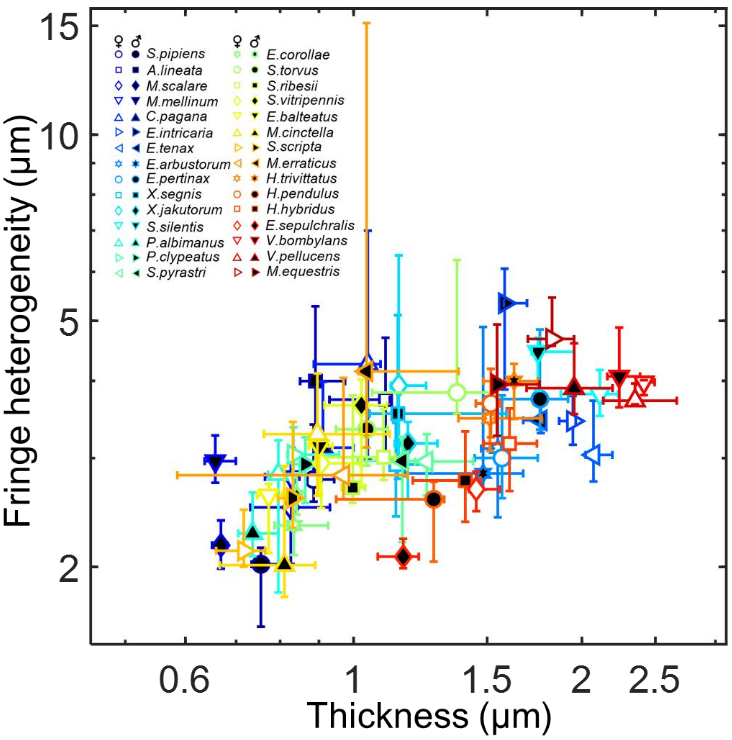

Figure S3: Scatter plot of the weighted medians and IQRs of the membrane thickness d_{wing} vs. the fringe heterogeneity λ_0. The species list is sorted and color-coded based on genetic similarity.

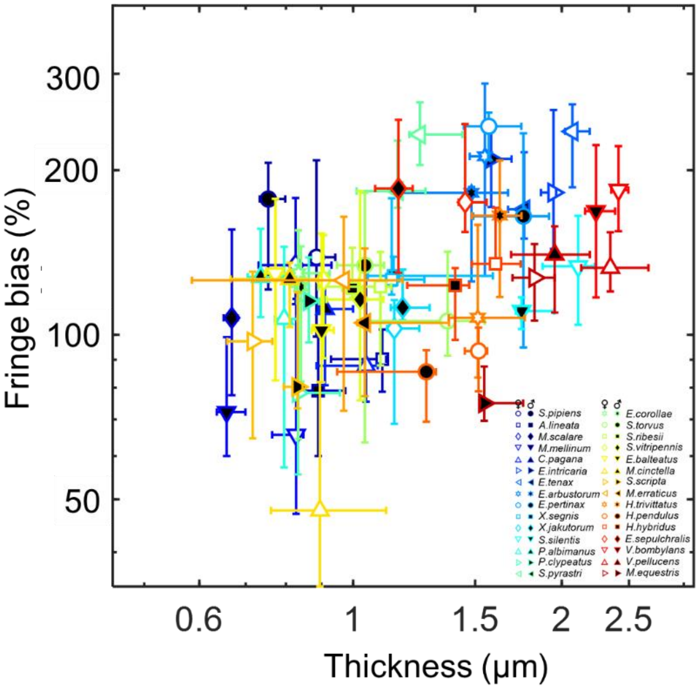

Figure S4: Scatter plot of the weighted medians and IQRs of the membrane thickness d_{wing} vs. the WIS bias β. The species list is sorted and color-coded based on genetic similarity.

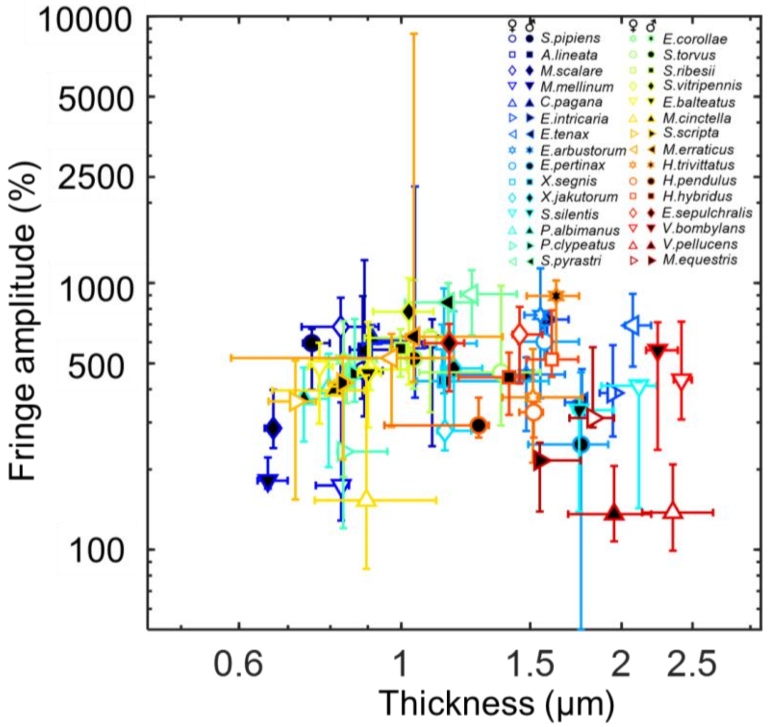

Figure S5: Scatter plot of the weighted medians and IQRs of the membrane thickness d_{wing} vs. the fringe amplitude α. The species list is sorted and color-coded based on genetic similarity.

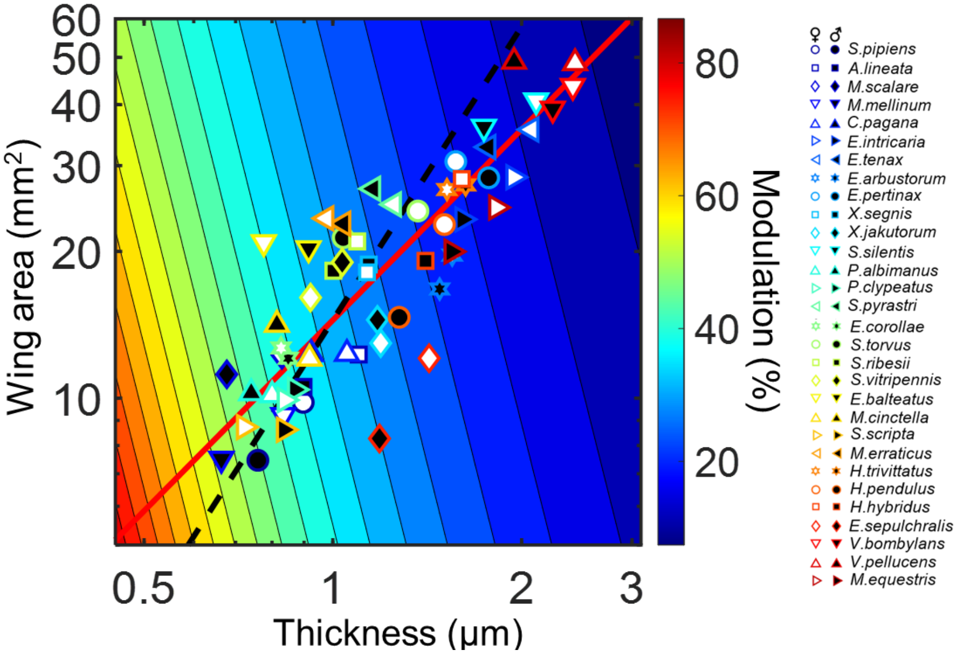

Figure S6: Scatter plot of the membrane thickness d_{wing} vs. the wing area A_{wing} vs. the modulation depth M.

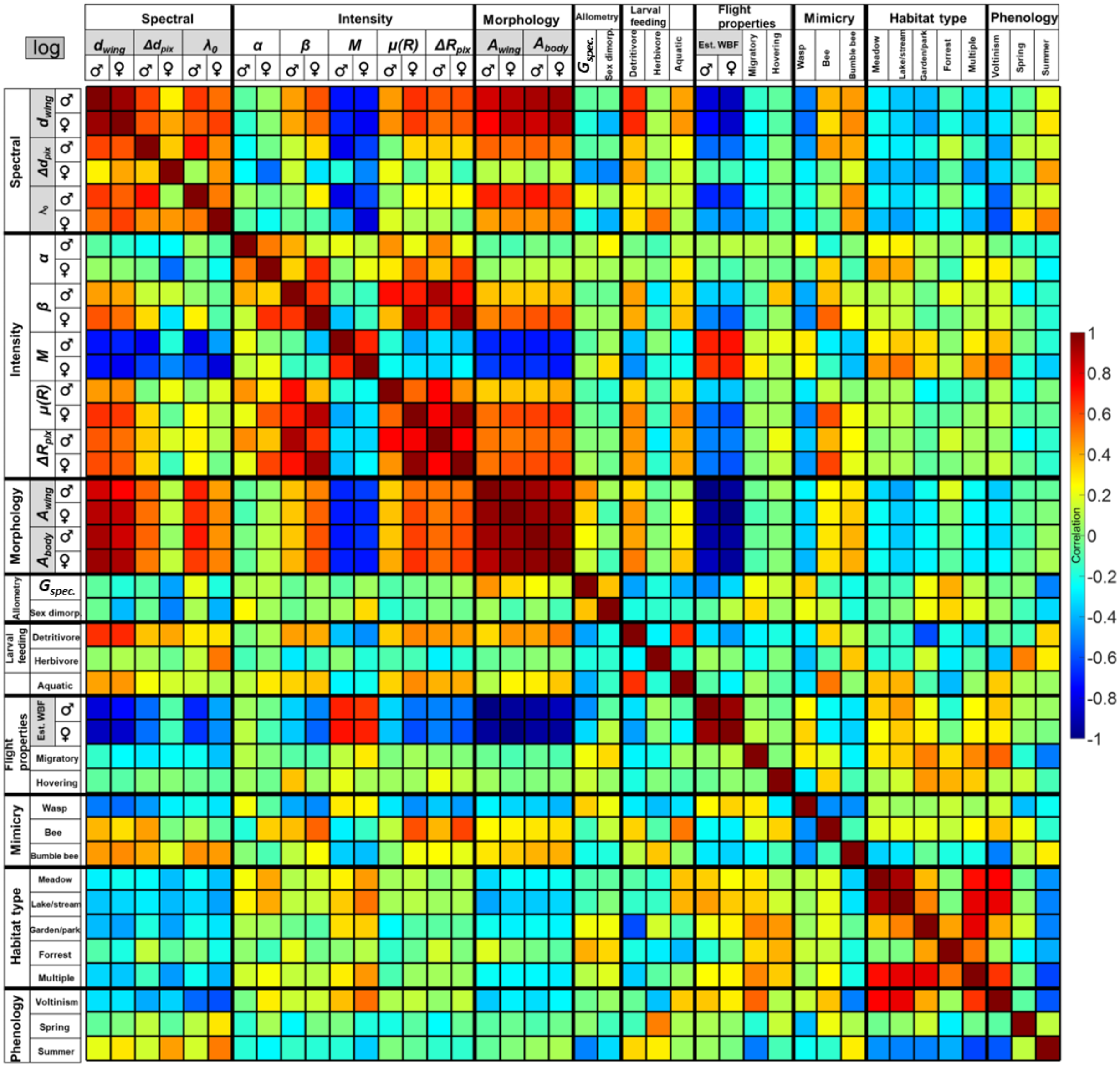

Figure S7: Correlation matrix of WIS parameters with ecological traits (full version). Definination of abbreviations; d_{wing}: membrane thickness, Δd_{pix}: wing heterogeneity, λ_0: fringe heterogeneity, α: effective fringe amplitude, β: effective fringe bias , M: fringe modulation depth, $\mu(R)$: broadband reflectance, ΔR_{pix}: degree of wrinkling , A_{wing}: wing area, A_{body}: body area, γ: allometric power relation, Est.WBF: estimated wing beat frequency.

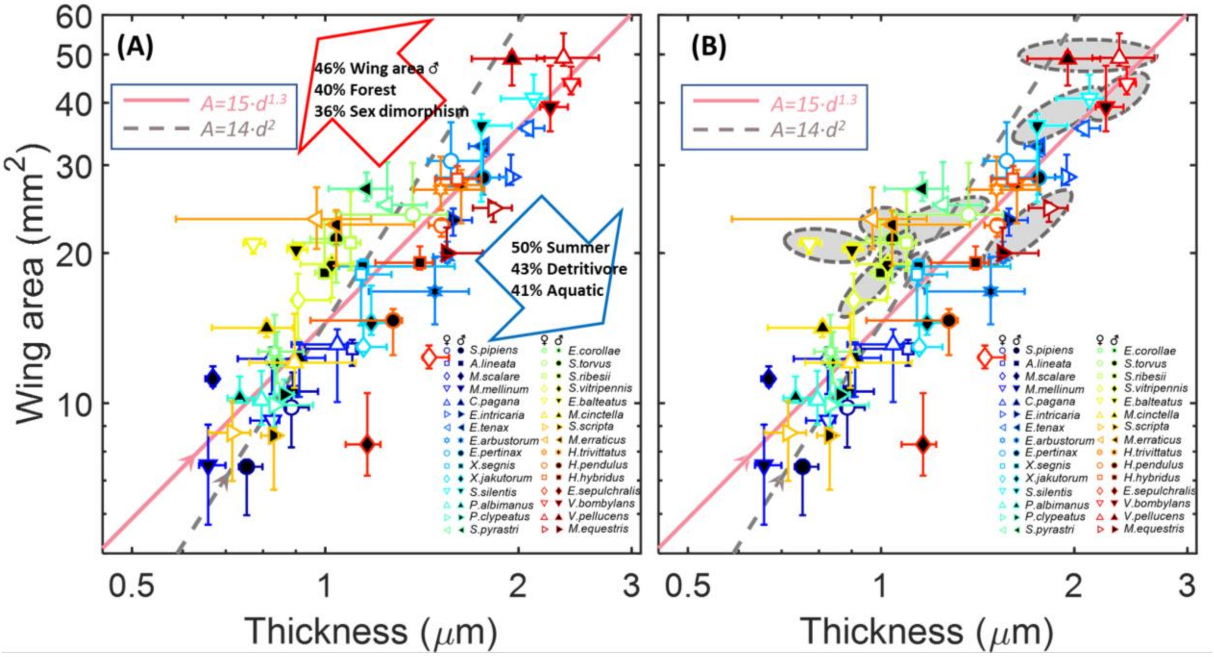

Figure S8: (A) Relationship between WIS features and ecological traits. (B) Species that display weak sexual dimorphism in body shape and size, according to Table 1, are indicated by gray circles.

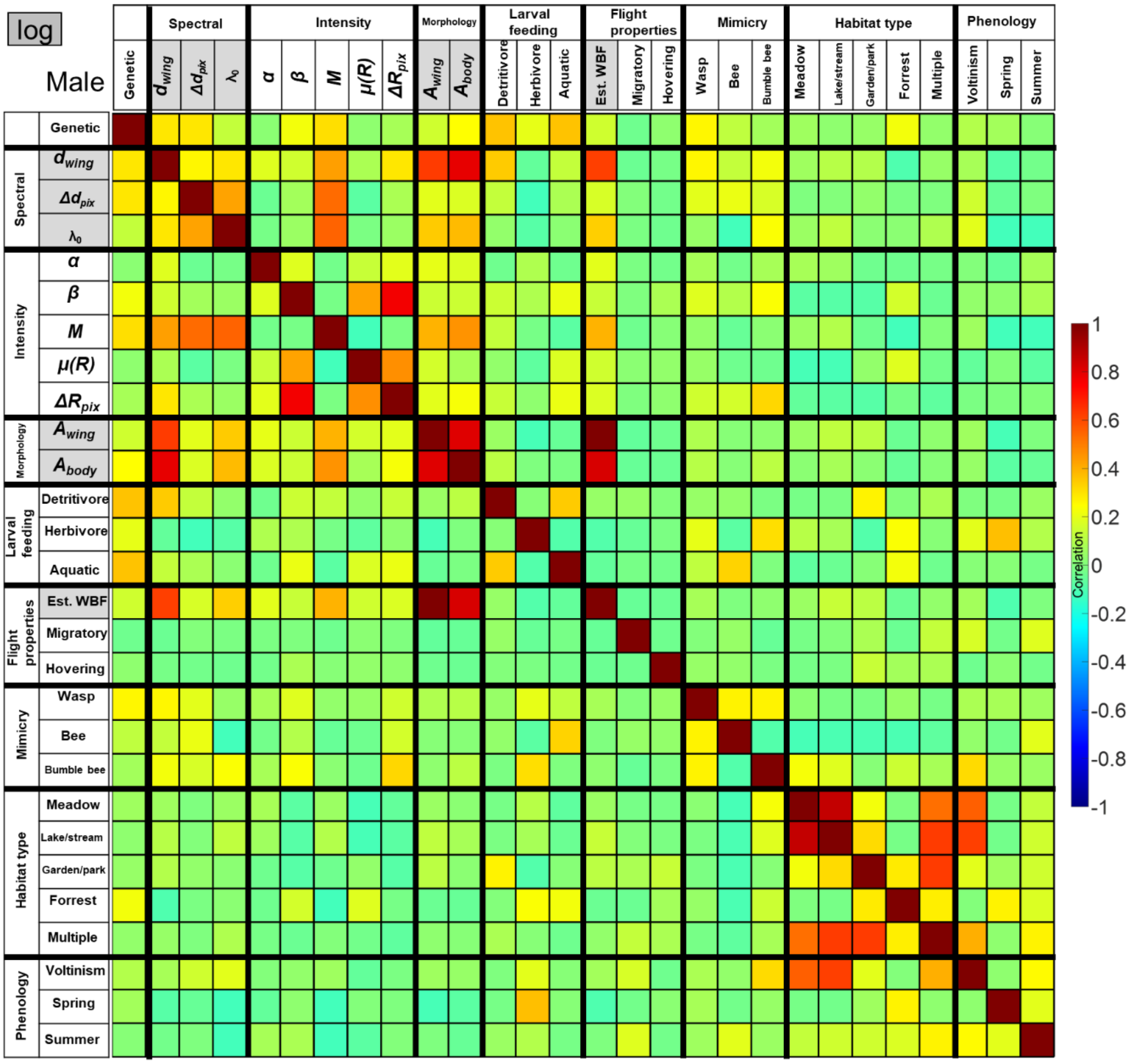

Figure S9: Correlation matrix of the pairwise distance between genetic markers, WIS parameters, and ecological traits for male hover flies. Definition of abbreviations; d_{wing}: membrane thickness, Δd_{pix}: wing heterogeneity, λ_0: fringe heterogeneity, α: effective fringe amplitude, β: effective fringe bias , M: fringe modulation depth, $\mu(R)$: broadband reflectance, ΔR_{pix}: degree of wrinkling , A_{wing}: wing area, A_{body}: body area, γ: allometric power relation, Est.WBF: estimated wing beat frequency.

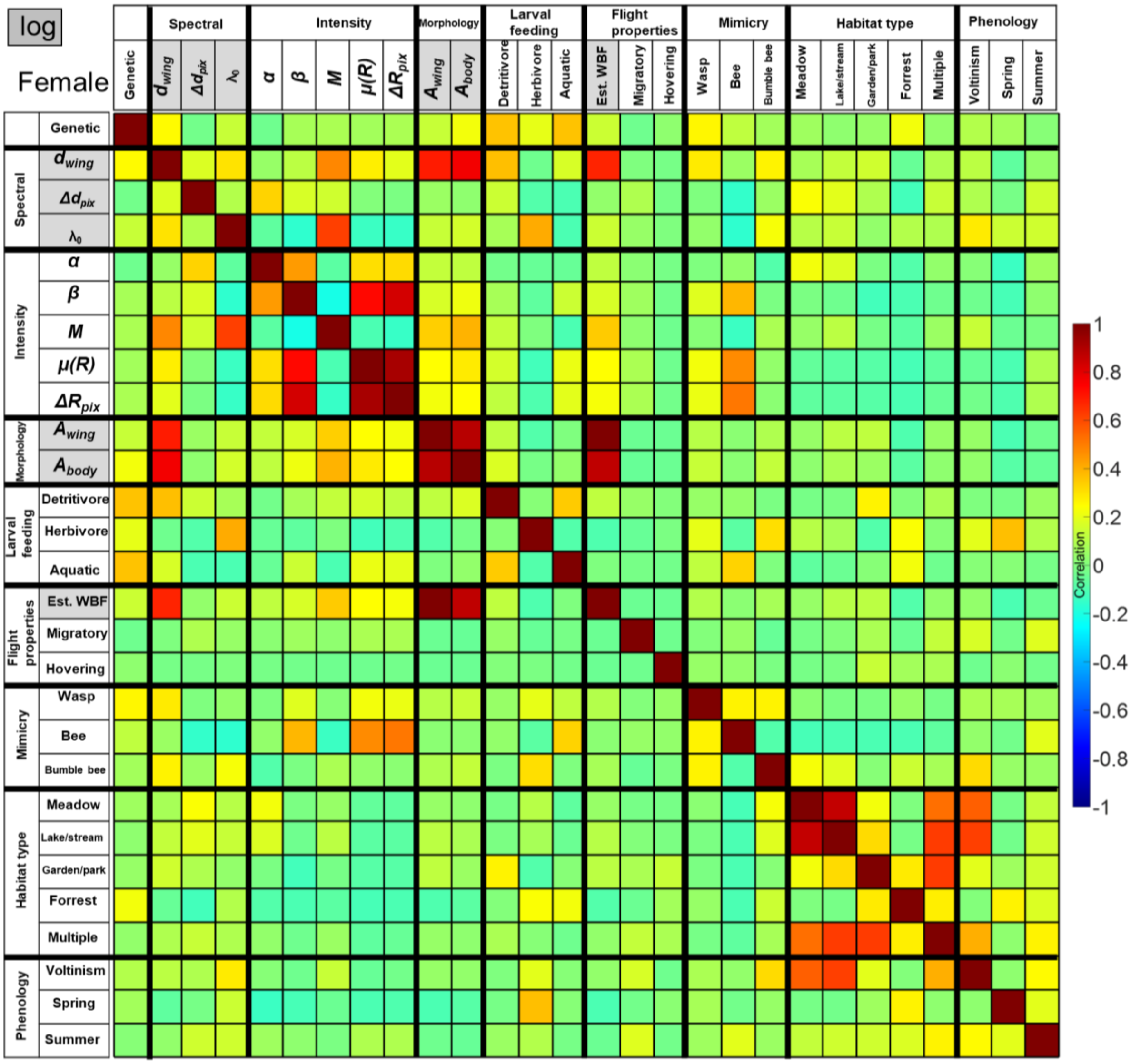

Figure S10: Correlation matrix of the pairwise distance between genetic markers, WIS parameters, and ecological traits for female hover flies. Definition of abbreviations; d_{wing}: membrane thickness, Δd_{pix}: wing heterogeneity, λ_0: fringe heterogeneity, α: effective fringe amplitude, β: effective fringe bias , M: fringe modulation depth, $\mu(R)$: broadband reflectance, ΔR_{pix}: degree of wrinkling , A_{wing}: wing area, A_{body}: body area, γ: allometric power relation, Est.WBF: estimated wing beat frequency.

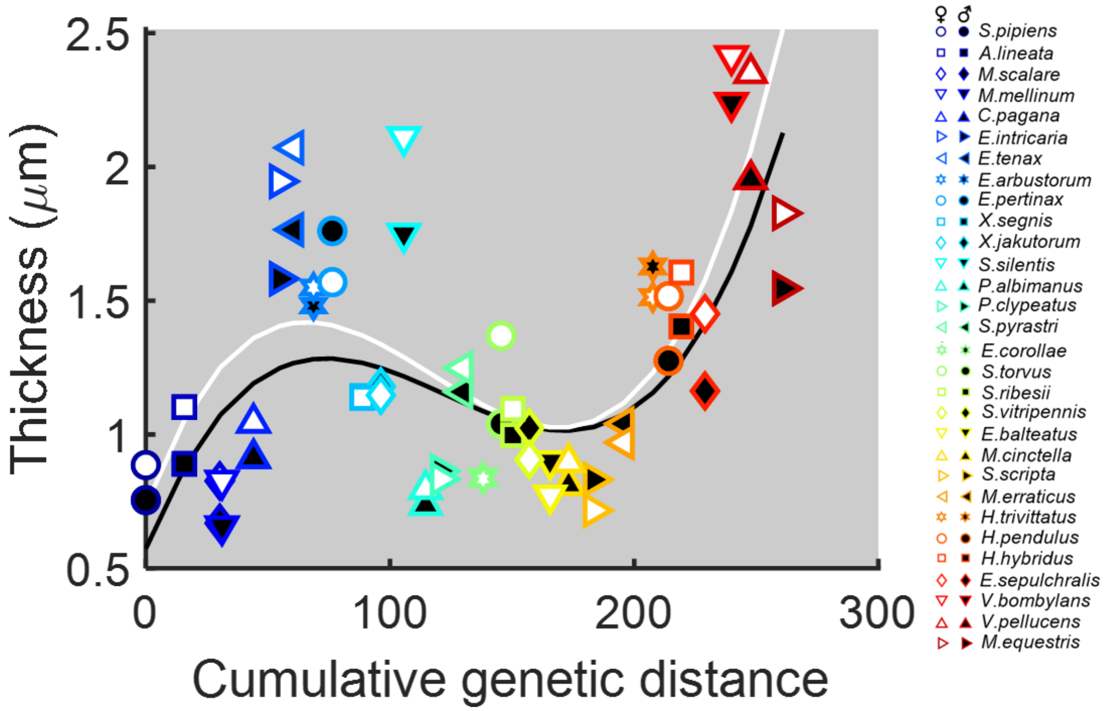

Figure S11: Wing thickness variation for male and females across the hover fly family. The thickness parameter folds twice across the family, this somewhat explains the moderate (linear) correlation between genetic distance and wing thickness differences.

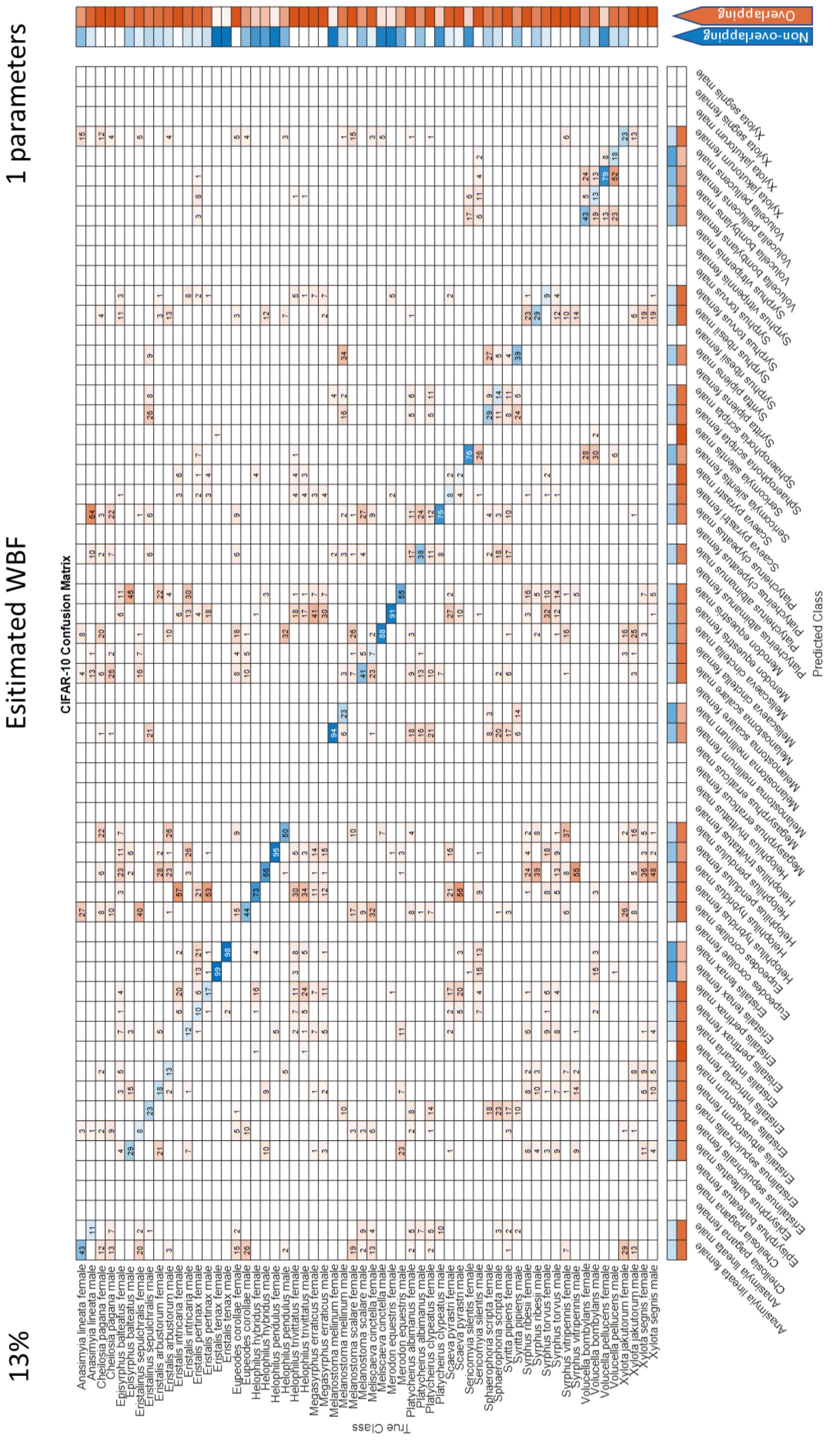

Figure S12: Confusion matrix and estimation of possible overlap between species based on estimated wing beat frequency $\hat{f}$.

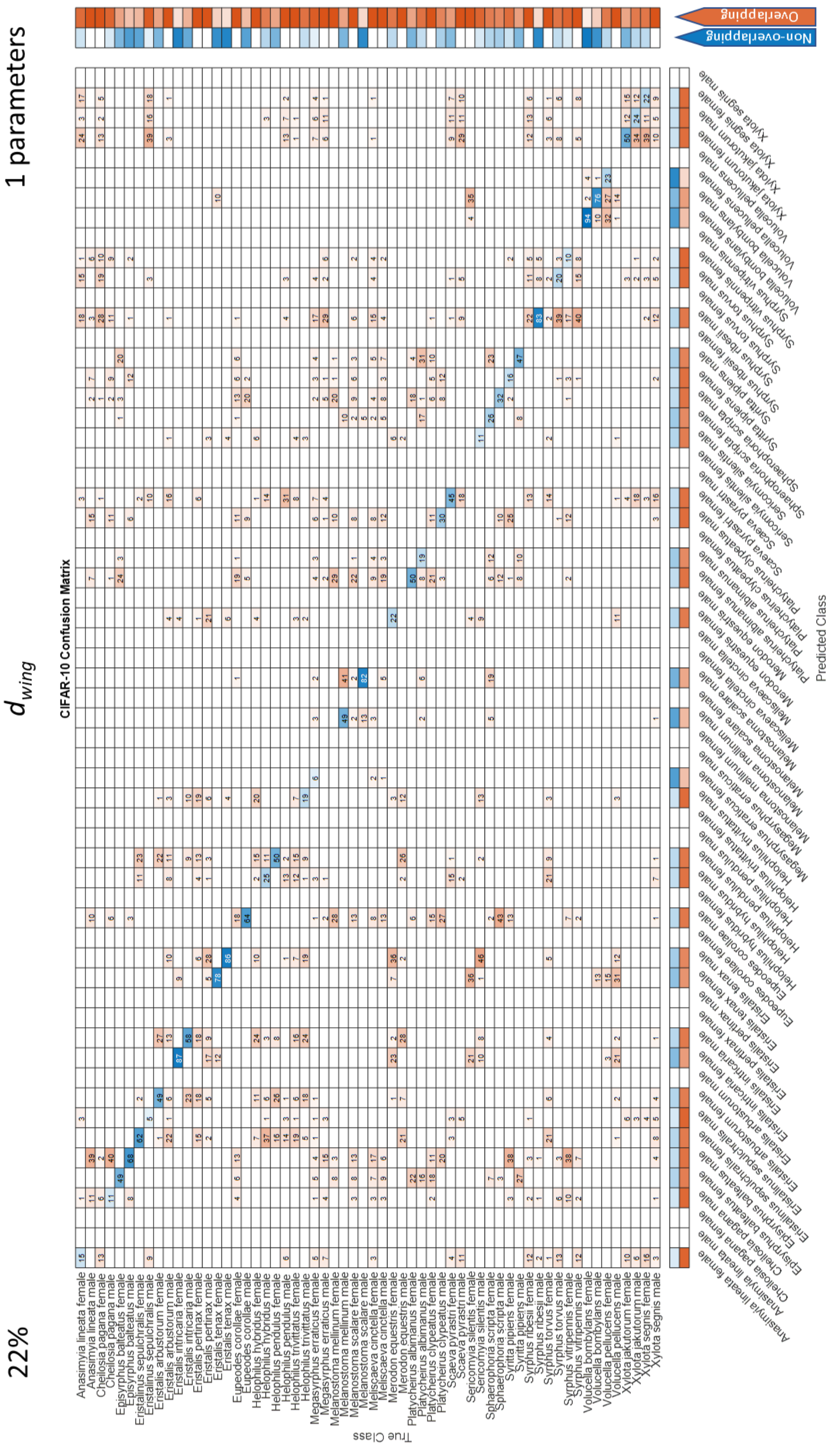

Figure S13: Confusion matrix and estimation of possible overlap between species based on the estimated membrane thickness d_{wing}.

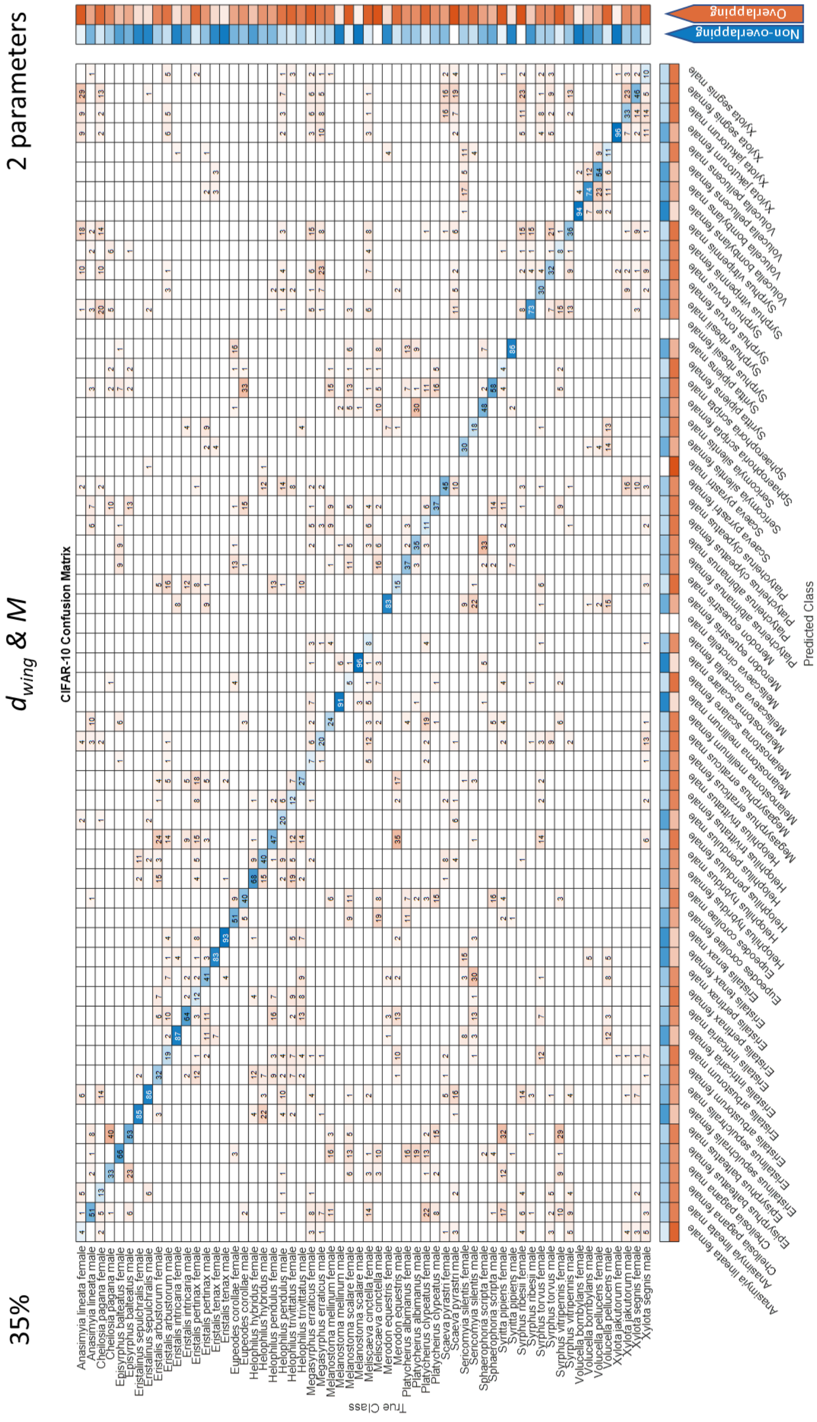

Figure S14: Confusion matrix and estimation of possible overlap between species based on the estimated membrane thickness d_{wing} and modulation depth M.

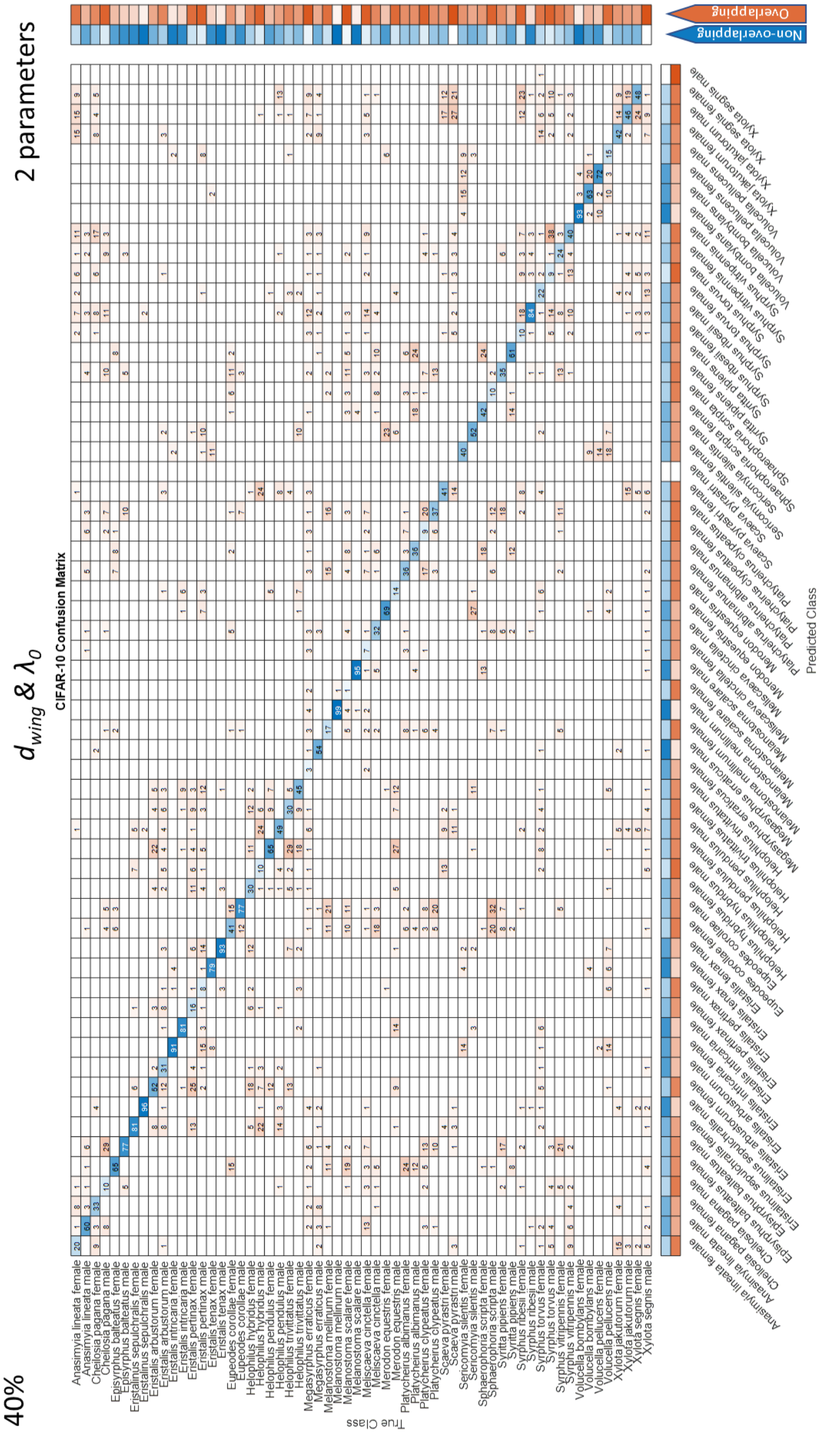

Figure S15: Confusion matrix and estimation of possible overlap between species based on the estimated membrane thickness d_{wing} and fringe heterogeneity λ_0.

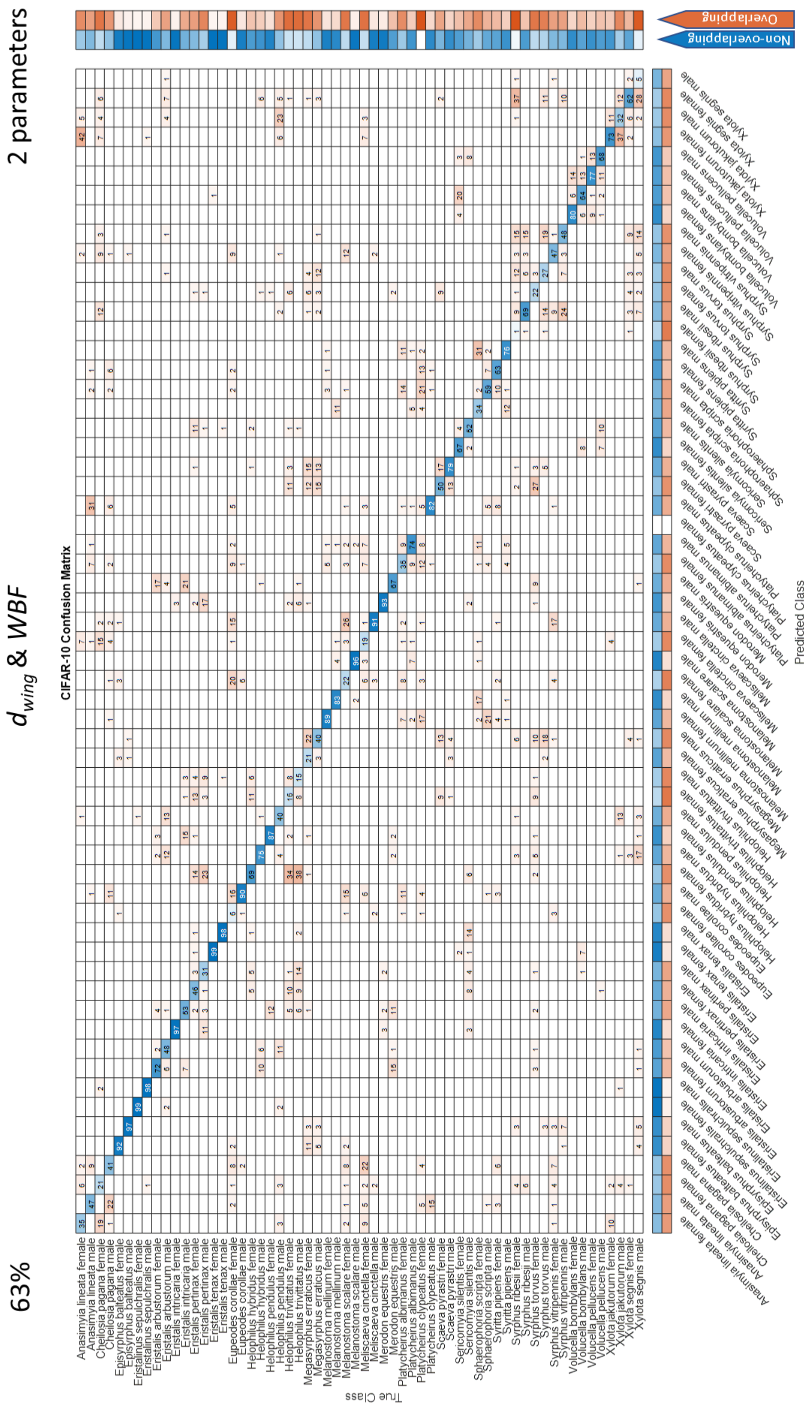

Figure S16: Confusion matrix and estimation of possible overlap between species based on the estimated membrane thickness d_{wing} and estimated wingbeat frequency $\hat{f}$.

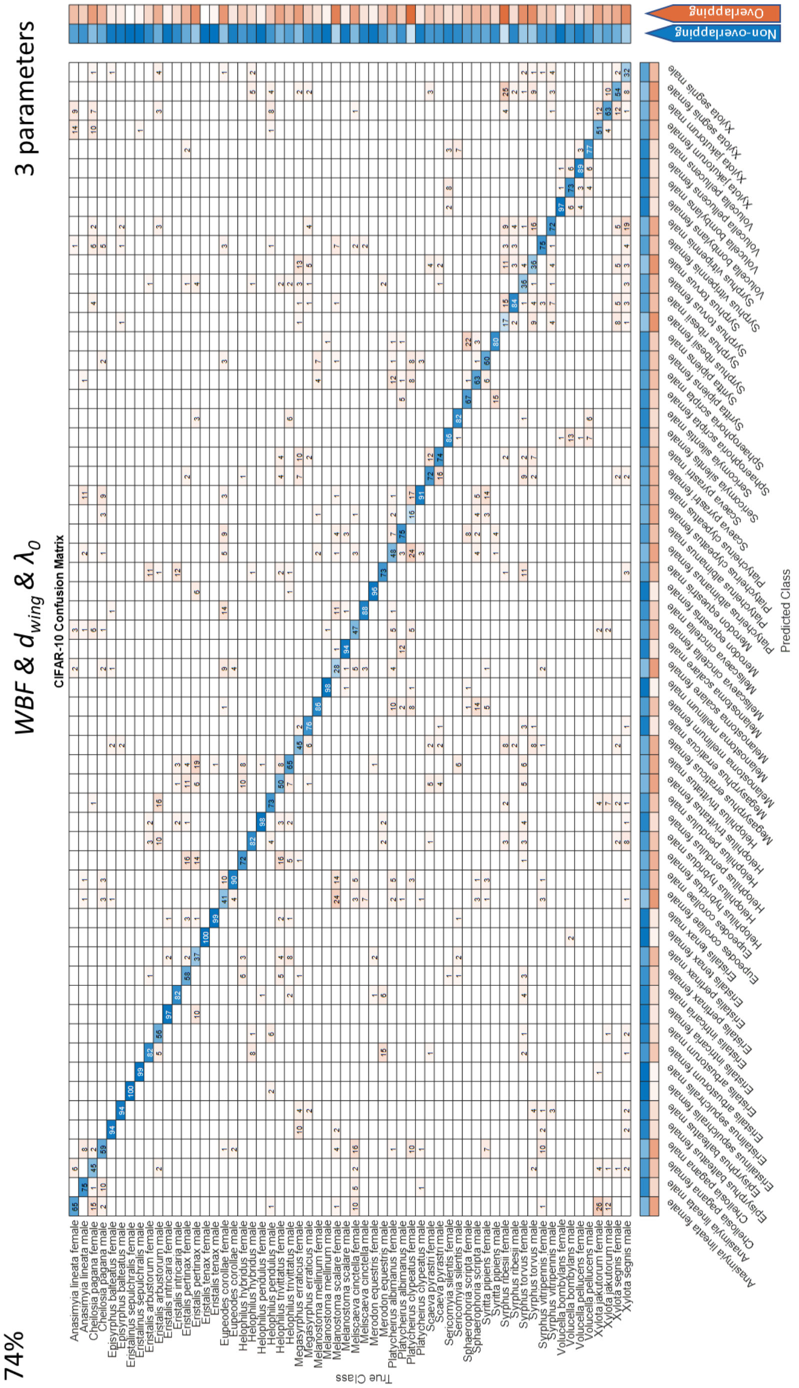

Figure S17: Confusion matrix and estimation of possible overlap between species based on the estimated wing beat frequency $\hat{f}$, estimated membrane thickness d_{wing} and fringe heterogeneity λ_0.

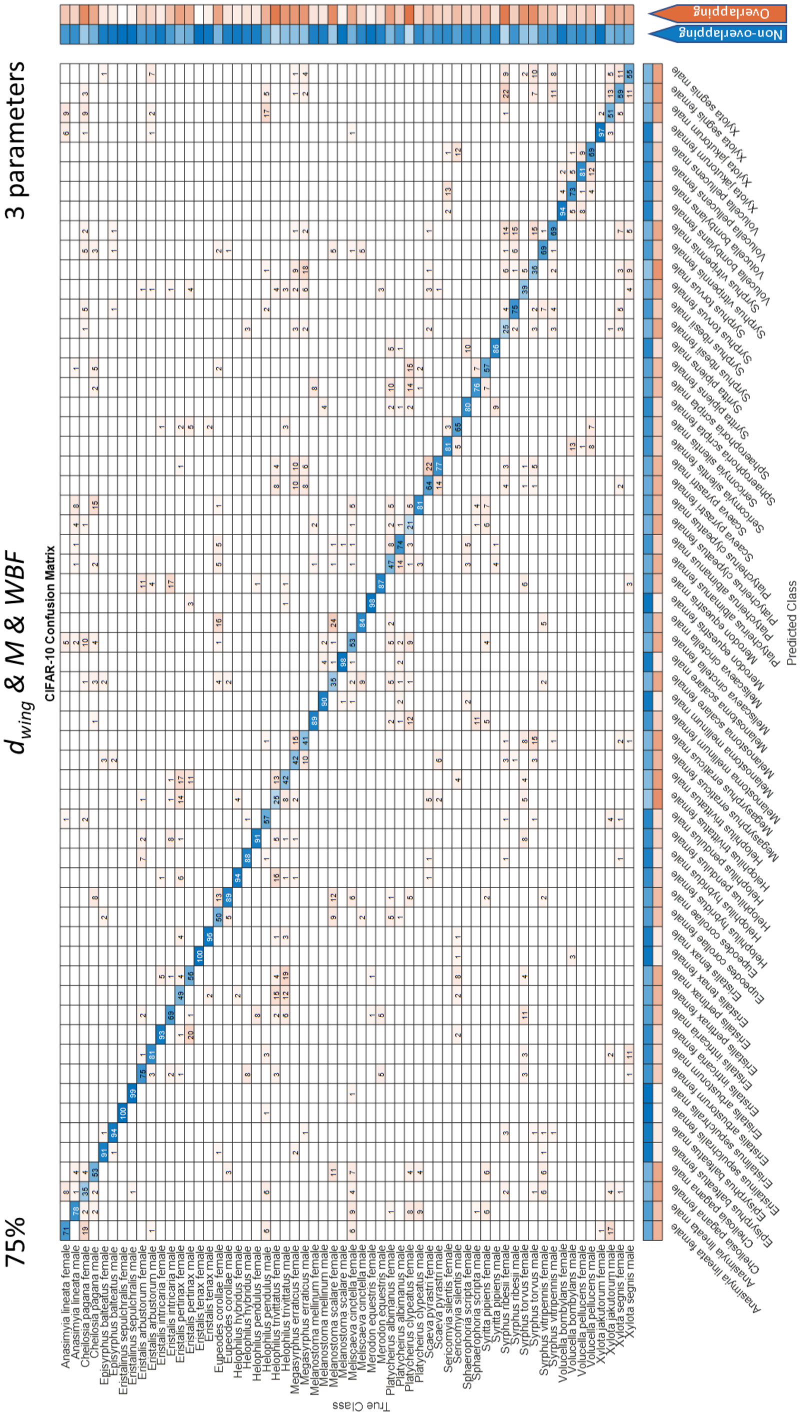

Figure S18: Confusion matrix and estimation of possible overlap between species based on the estimated membrane thickness d_{wing}, modulation depth M and estimated wing beat frequency $\hat{f}$.

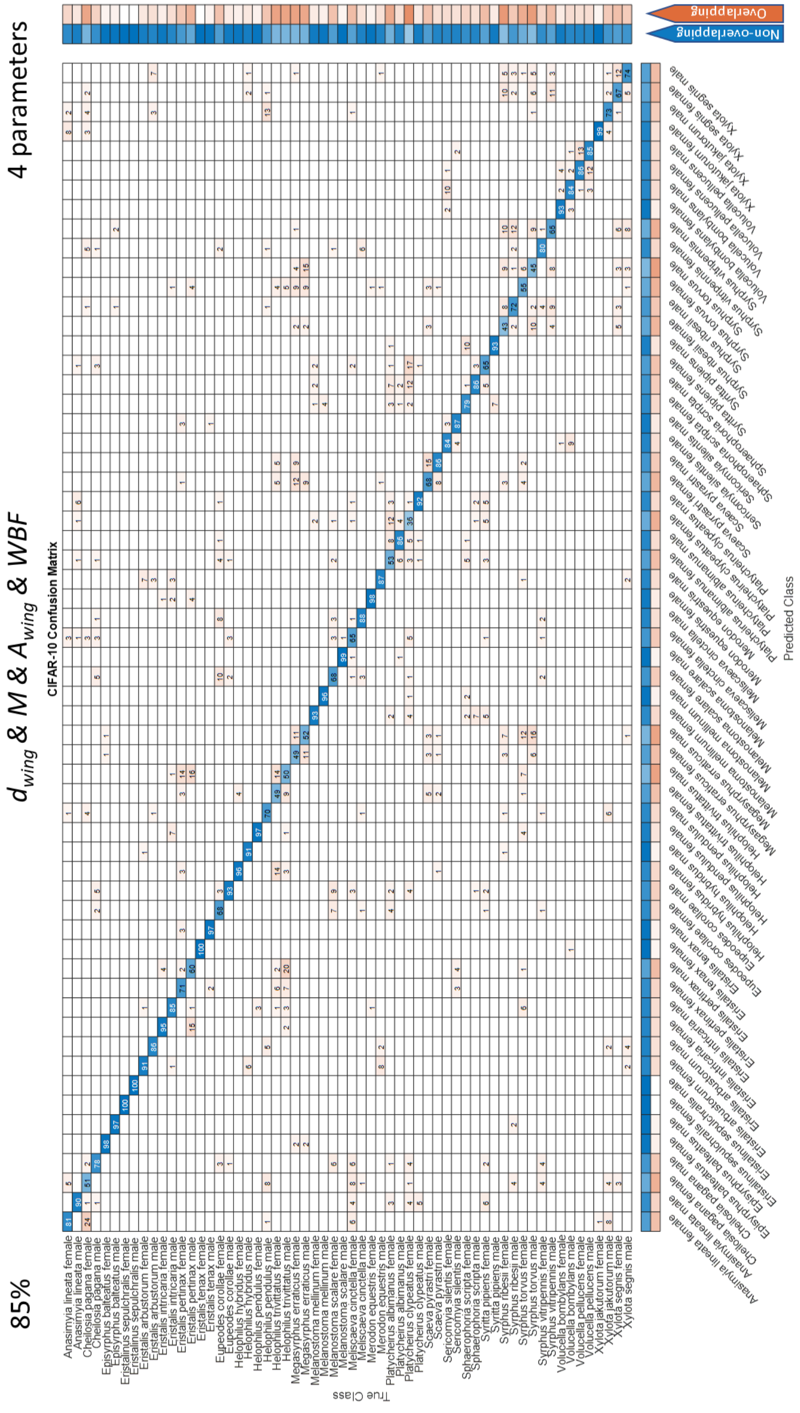

Figure S19: Confusion matrix and estimation of possible overlap between species based on the estimated membrane thickness d_{wing}, modulation depth M, wing area A, and estimated wing beat frequency $\hat{f}$.

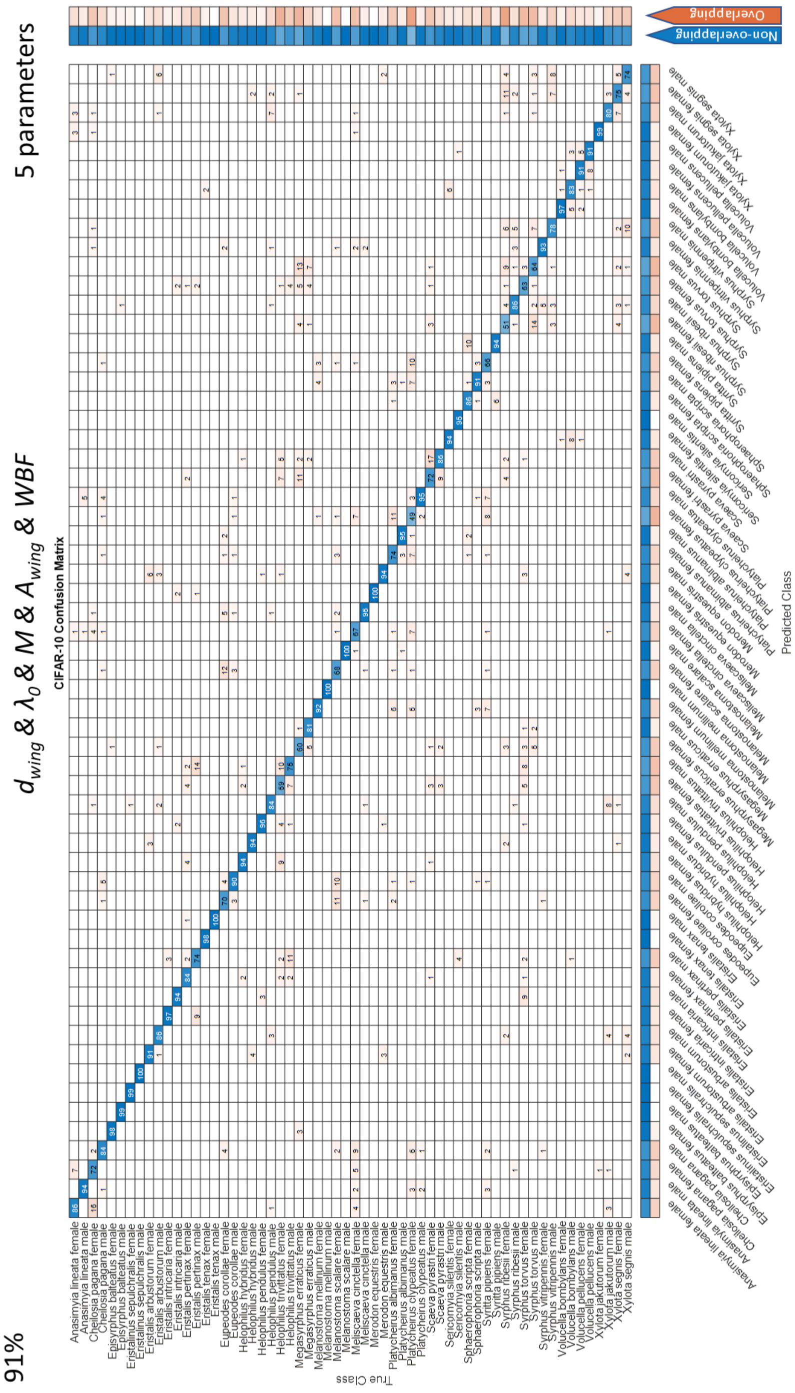

Figure S20: Confusion matrix and estimation of possible overlap between species based on the estimated membrane thickness d_{wing}, modulation depth M, wing area A, and estimated wing beat frequency $\hat{f}$.

Supplementary Tables:

Table S1: The 30 studied species of insect pollinators from the family Syrphdae. Species that display weak sexual dimorphism according to the literature are highlighted in gray.

Genus	Species	Author, year	Genus	Species	Author, year
Anasimyia	*lineata*	(Fabricius, 1787)	*Meliscaeva*	*cinctella*	(Zetterstedt, 1843)
Cheilosia	*pagana*	(Meigen, 1822)	*Merodon*	*equestris*	(Fabricius, 1794)
Episyrphus	*balteatus*	(De Geer, 1776)	*Platycheirus*	*albimanus*	(Fabricius, 1781)
Eristalinus	*sepulchralis*	(Linnaeus, 1758)		*clypeatus*	(Meigen, 1822)
Eristalis	*arbustorum*	(Linnaeus, 1758)	*Scaeva*	*pyrastri*	(Linnaeus, 1758)
	intricaria	(Linnaeus, 1758)	*Sericomyia*	*silentis*	(Harris, 1776)
	pertinax	(Scopoli, 1763)	*Sphaerophoria*	*scripta*	(Linnaeus, 1758)
	tenax	(Linnaeus, 1758)	*Syritta*	*pipiens*	(Linnaeus, 1758)
Eupeodes	*corollae*	(Fabricius, 1794)	*Syrphus*	*ribesii*	(Linnaeus, 1758)
Helophilus	*pendulus*	(Linnaeus, 1758)		*torvus*	Osten Sacken, 1875
	trivittatus	(Fabricius, 1805)		*vitripennis*	Meigen, 1822
	hybridus	Loew, 1846	*Volucella*	*bombylans*	(Linnaeus, 1758)
Megasyrphus	*erraticus*	(Linnaeus, 1758)		*pellucens*	(Linnaeus, 1758)
Melanostoma	*mellinum*	(Linnaeus, 1758)	*Xylota*	*jakutorum*	Bagachanova, 1980
	scalare	(Fabricius, 1794)		*segnis*	(Linnaeus, 1758)

Table S2: All parameterized parameters from spectral fringe and hyperspectral data.

Male	$F(\lambda,d)=\frac{4r^2\sin^2\left(2\pi d\sqrt{n^2-\sin^2\theta}/\lambda\right)}{(1-r^2)^2+4r^2\sin^2\left(2\pi d\sqrt{n^2-\sin^2\theta}/\lambda\right)}$ $F_{eff}=\frac{Amp\cdot F_{pix(d)}\cdot\lambda^{\alpha}+Bias\cdot\lambda_0^{\alpha}}{(\lambda_0^{\alpha}+\lambda^{\alpha})}$, $\alpha=2.71$					$M=\frac{\sigma(R_\lambda)\cdot\mu(F_\lambda)}{\sigma(F_\lambda)\cdot\mu(R_\lambda)}$				
**Species**	Thickness d (µm)	Amplitude *α*	Bias *β*	λ_0 (µm)	R^2	Modulation *M* (%)	Mean R	Wrinkle Δ(R)	Heterogeneity Δ(D) (µm)	Wing area A (mm^2)
S.pipiens	0.75 (0.73-0.8)	6 (4-6.7)	1.8 (1.2-2.1)	2 (1.6-2.1)	0.99	72 (59-85)	0.21 (0.14-0.28)	0.44 (0.24-0.57)	0.58 (0.49-0.79)	7.5 (6-7.7)
A.lineata	0.89 (0.85-0.97)	5.6 (3.2-12.2)	0.8 (0.6-1)	4 (2.8-5.3)	0.97	29 (20-37)	0.18 (0.13-0.25)	0.26 (0.13-0.31)	0.73 (0.67-0.83)	10.6 (10.4-11.5)
M.scalare	0.67 (0.65-0.68)	2.9 (2.4-4)	1.1 (0.8-1.6)	2.2 (2-2.4)	0.99	39 (32-42)	0.17 (0.15-0.23)	0.24 (0.14-0.33)	0.67 (0.59-0.77)	11.2 (10.8-11.9)
M.mellinum	0.66 (0.64-0.7)	1.8 (1.7-2.2)	0.7 (0.6-1)	3 (2.7-3.3)	0.99	17 (17-60)	0.14 (0.09-0.19)	0.16 (0.12-0.36)	0.71 (0.66-0.99)	7.5 (5.7-9.1)
C.pagana	0.91 (0.89-1)	6.2 (4.7-7)	1.1 (0.8-1.2)	3.1 (2.9-4)	0.98	44 (31-45)	0.25 (0.12-0.28)	0.31 (0.21-0.38)	0.7 (0.68-0.78)	12.3 (10.3-13.2)
E.intricaria	1.58 (1.58-1.69)	7.3 (5.3-7.8)	2.1 (1.7-2.4)	5.3 (3.9-6.1)	0.88	13 (8-22)	0.24 (0.23-0.35)	0.72 (0.62-0.91)	0.82 (0.79-0.89)	23.3 (21.7-24.6)
E.tenax	1.77 (1.67-1.77)	3.6 (3.2-4.8)	1.7 (1.5-2.2)	3.5 (3.3-3.7)	0.94	21 (18-24)	0.27 (0.24-0.34)	0.57 (0.42-0.73)	0.76 (0.75-0.78)	32.7 (31.2-33.4)
E.arbustorum	1.48 (1.17-1.67)	4.5 (2.8-5.3)	1.8 (1.3-1.9)	2.8 (2.7-4.9)	0.77	16 (16-19)	0.31 (0.22-0.36)	0.65 (0.44-0.68)	0.75 (0.7-0.87)	16.7 (14.4-19.6)
E.pertinax	1.76 (1.49-1.92)	2.5 (0.5-4.6)	1.6 (0.9-2.3)	3.7 (3.3-4.8)	0.88	14 (9-15)	0.19 (0.18-0.47)	0.56 (0.45-0.9)	0.85 (0.81-0.96)	28.3 (26.2-32.7)
X.segnis	1.14 (1.05-1.59)	4.3 (3.9-9.5)	1.3 (1.2-1.4)	3.5 (2.8-5.1)	0.92	19 (14-21)	0.24 (0.17-0.28)	0.48 (0.46-0.61)	0.77 (0.71-0.79)	18.8 (17.2-19.4)
X.jakutorum	1.18 (1.13-1.29)	4.8 (2.8-7.8)	1.1 (1.1-1.3)	3.2 (2.6-3.4)	0.95	23 (15-28)	0.19 (0.16-0.23)	0.36 (0.3-0.43)	0.74 (0.72-0.76)	14.5 (13.7-17.2)
S.silentis	1.75 (1.72-1.95)	3.3 (1.4-3.7)	1.1 (1-1.2)	4.5 (3.9-4.7)	0.87	12 (8-17)	0.22 (0.16-0.23)	0.31 (0.3-0.42)	0.74 (0.71-0.81)	36 (25.3-38)
P.albimanus	0.74 (0.7-0.8)	3.7 (2.5-4.8)	1.3 (1.1-1.6)	2.3 (2.1-2.6)	0.99	50 (27-55)	0.2 (0.13-0.26)	0.3 (0.12-0.39)	0.68 (0.62-0.76)	10.2 (10.1-11.3)
P.clypeatus	0.86 (0.83-0.89)	4.5 (3.6-7.3)	1.2 (1-1.4)	2.9 (2.5-3.2)	0.99	43 (33-49)	0.19 (0.12-0.22)	0.3 (0.24-0.35)	0.7 (0.69-0.72)	10.4 (10-11.1)
S.pyrastri	1.16 (1.01-1.27)	8.5 (6.5-10)	1.8 (1.7-2.3)	3 (2.2-3.2)	0.92	29 (21-43)	0.3 (0.28-0.4)	0.67 (0.61-0.89)	0.7 (0.65-0.7)	26.9 (25.4-29)
E.corollae	0.84 (0.81-0.85)	4.2 (3.5-5.4)	1.2 (0.8-1.5)	2.6 (2.4-2.8)	0.99	47 (39-58)	0.2 (0.12-0.26)	0.34 (0.2-0.38)	0.61 (0.53-0.68)	12.1 (11-13.9)
S.torvus	1.04 (0.95-1.11)	5.2 (4-10.2)	1.3 (0.6-1.8)	3.3 (2.9-4.1)	0.96	21 (17-39)	0.31 (0.23-0.46)	0.53 (0.37-0.7)	0.74 (0.68-0.76)	21.4 (17.4-24.1)
S.ribesii	1 (0.96-1.04)	5.7 (4.5-6.7)	1.2 (1.2-1.4)	2.7 (2.5-3.8)	0.99	36 (24-46)	0.26 (0.23-0.28)	0.44 (0.34-0.58)	0.67 (0.65-0.7)	18.3 (17.8-23.3)
S.vitripennis	1.02 (0.92-1.11)	7.8 (4.2-10.4)	1.2 (0.8-1.8)	3.7 (2.7-4)	0.98	27 (20-32)	0.25 (0.19-0.45)	0.35 (0.22-0.78)	0.65 (0.63-0.71)	19 (16.3-19.4)
E.balteatus	0.9 (0.87-0.94)	4.6 (2.9-7.1)	1 (0.8-1.5)	3.1 (2.6-3.2)	0.49	41 (36-46)	0.18 (0.1-0.29)	0.31 (0.19-0.5)	0.64 (0.59-0.74)	20.3 (19.5-20.9)
M.cinctella	0.81 (0.67-0.89)	4 (3.8-4.1)	1.3 (1-1.4)	2 (1.8-2.9)	0.99	56 (36-65)	0.23 (0.21-0.25)	0.4 (0.31-0.46)	0.64 (0.62-0.7)	14.2 (13.6-14.6)
S.scripta	0.83 (0.79-0.85)	4.2 (2.2-5.3)	0.8 (0.7-1.2)	2.6 (2.3-3.4)	0.98	43 (36-49)	0.13 (0.11-0.17)	0.22 (0.15-0.29)	0.69 (0.6-0.73)	8.6 (6.7-8.9)
M.erraticus	1.04 (1.02-1.38)	6.3 (4.3-86.1)	1.1 (0.8-1.4)	4.1 (3.1-15.1)	0.92	18 (9-27)	0.18 (0.16-0.27)	0.35 (0.22-0.48)	0.68 (0.65-0.72)	22.8 (18.8-24.4)
H.trivittatus	1.63 (1.48-1.75)	8.9 (5.3-10.2)	1.7 (1.2-2.1)	4 (3.5-4.3)	0.93	18 (15-26)	0.39 (0.22-0.53)	0.64 (0.41-0.84)	0.7 (0.69-0.75)	27.4 (22.9-29.4)
H.pendulus	1.28 (0.95-1.32)	2.9 (2.6-3.7)	0.9 (0.7-0.9)	2.6 (2-2.7)	0.99	30 (24-56)	0.15 (0.14-0.25)	0.22 (0.17-0.26)	0.67 (0.61-0.71)	14.7 (12.5-15.4)
H.hybridus	1.4 (1.2-1.47)	4.4 (3.2-5.5)	1.2 (1-1.3)	2.8 (2.4-3.3)	0.98	32 (28-36)	0.28 (0.23-0.29)	0.42 (0.34-0.45)	0.65 (0.62-0.72)	19.1 (18.5-20.6)
E.sepulchralis	1.16 (1.08-1.22)	6 (3.9-7)	1.9 (1.3-2.5)	2.1 (2-2.2)	0.99	37 (34-40)	0.41 (0.3-0.42)	0.52 (0.36-0.76)	0.62 (0.58-0.65)	8.3 (7.2-10.5)
V.bombylans	2.24 (2.16-2.39)	5.6 (2.4-7.1)	1.7 (1.2-2.2)	4.1 (3.6-4.9)	0.88	13 (8-14)	0.31 (0.24-0.47)	0.68 (0.39-0.91)	0.78 (0.78-0.81)	39.2 (35.1-47.5)
V.pellucens	1.95 (1.69-2.2)	1.4 (1.1-2.1)	1.4 (1.1-1.6)	3.9 (3.5-4.6)	0.91	10 (8-18)	0.27 (0.21-0.35)	0.42 (0.28-0.56)	0.75 (0.73-0.8)	49 (43.3-50.5)
M.equestris	1.55 (1.52-1.76)	2.2 (1.4-2.5)	0.7 (0.7-0.9)	3.9 (3.3-4.9)	0.97	17 (10-18)	0.16 (0.14-0.19)	0.18 (0.17-0.23)	0.74 (0.72-0.82)	20 (19.8-22.5)

Table S3: All parameterized parameters from spectral fringe and hyperspectral data.

**Female**	$F(\lambda,d)=\frac{4r^2\sin^2\left(2\pi d\sqrt{n^2-\sin^2\theta}/\lambda\right)}{(1-r^2)^2+4r^2\sin^2\left(2\pi d\sqrt{n^2-\sin^2\theta}/\lambda\right)}$ $F_{eff}=\frac{Amp\cdot F_{pix(d)}\cdot\lambda^{\alpha}+Bias\cdot\lambda_0^{\alpha}}{(\lambda_0^{\alpha}+\lambda^{\alpha})}$, $\alpha=2.71$					$M=\frac{\sigma(R_\lambda)\cdot\mu(F_\lambda)}{\sigma(F_\lambda)\cdot\mu(R_\lambda)}$				
**species**	Thickness d (µm)	Amplitude *α*	Bias *β*	λ_0 (µm)	R^2	Modulation *M* (%)	Gradient *g*	Wrinkle Δ(R)	Heterogeneity Δ(D) (µm)	Wing area A (mm^2)
S.pipiens	0.89 (0.87-0.94)	4.7 (3.7-8.9)	1.4 (1-2.1)	2.8 (2.6-3.4)	0.98	41 (35-60)	0.27 (0.18-0.37)	0.54 (0.37-0.8)	0.67 (0.59-0.7)	9.8 (8.2-10.4)
A.lineata	1.1 (0.93-1.12)	6 (2.4-7.3)	0.9 (0.8-1)	3.7 (3-4.7)	0.98	26 (14-32)	0.2 (0.17-0.27)	0.26 (0.19-0.3)	0.74 (0.68-0.79)	12.9 (11.9-13.4)
M.scalare	0.83 (0.73-0.93)	6.8 (4-8.8)	1.3 (1.1-1.8)	2.5 (2-2.9)	0.99	52 (44-68)	0.22 (0.15-0.27)	0.37 (0.32-0.46)	0.66 (0.55-0.8)	12.3 (10.4-12.9)
M.mellinum	0.83 (0.76-0.85)	1.7 (1.3-3.6)	0.7 (0.5-1.2)	2.8 (2.4-3.1)	0.99	35 (28-54)	0.1 (0.07-0.31)	0.12 (0.07-0.47)	0.75 (0.7-0.92)	9.2 (8.7-9.9)
C.pagana	1.04 (0.89-1.09)	6 (3.7-23)	0.9 (0.8-1.4)	4.3 (3-7)	0.97	29 (19-39)	0.18 (0.11-0.29)	0.26 (0.22-0.47)	0.72 (0.68-0.74)	13.1 (10.1-14.1)
E.intricaria	1.95 (1.87-1.96)	3.9 (2.7-5.8)	1.8 (1.4-2.6)	3.4 (3.2-3.8)	0.97	16 (14-25)	0.36 (0.15-0.44)	0.69 (0.39-0.82)	0.75 (0.69-0.87)	28.4 (27.4-31.5)
E.tenax	2.07 (2.02-2.19)	6.9 (4.9-9.1)	2.4 (1.9-2.6)	3 (2.8-3.7)	0.94	21 (17-29)	0.47 (0.28-0.6)	0.95 (0.73-1.18)	0.72 (0.66-0.73)	35.6 (34.4-36.7)
E.arbustorum	1.55 (1.47-1.57)	7.6 (3.7-11.4)	2.1 (1.3-2.9)	3.1 (2.4-3.7)	0.96	27 (18-42)	0.56 (0.34-0.64)	0.95 (0.48-1.21)	0.69 (0.68-0.73)	19.7 (19.1-21.1)
E.pertinax	1.57 (1.44-1.75)	6 (5.2-6.9)	2.4 (2.1-2.5)	3 (2.6-3.8)	0.96	21 (15-27)	0.37 (0.32-0.39)	0.78 (0.7-0.9)	0.67 (0.63-0.76)	30.6 (27.2-36.6)
X.segnis	1.14 (1.12-1.27)	5.9 (4.1-7)	1.3 (1.2-1.8)	2.9 (2.4-3.4)	0.94	26 (17-29)	0.22 (0.2-0.38)	0.47 (0.43-0.6)	0.71 (0.68-0.78)	18.1 (14.3-18.7)
X.jakutorum	1.15 (1.12-1.25)	2.8 (2.4-5.6)	1 (0.7-1.2)	3.9 (2.9-6.4)	0.94	15 (14-17)	0.16 (0.13-0.2)	0.26 (0.19-0.39)	0.72 (0.66-0.78)	13 (12.7-14.5)
S.silentis	2.11 (1.88-2.23)	4.1 (1.4-4.3)	1.3 (1-1.6)	3.8 (3.5-4.2)	0.90	12 (10-24)	0.26 (0.22-0.32)	0.48 (0.25-0.52)	0.71 (0.69-0.73)	40.8 (40.6-45.5)
P.albimanus	0.8 (0.77-0.83)	4 (2-5.4)	1.1 (0.6-1.4)	2.8 (1.8-3.2)	0.99	55 (29-70)	0.16 (0.08-0.23)	0.28 (0.12-0.41)	0.67 (0.62-0.8)	10.1 (9.1-11.6)
P.clypeatus	0.83 (0.82-0.96)	2.3 (1.2-5.2)	0.8 (0.6-1.3)	3 (2.5-3.4)	0.99	33 (25-52)	0.11 (0.06-0.23)	0.17 (0.08-0.35)	0.71 (0.65-0.86)	9.9 (9-12.7)
S.pyrastri	1.25 (1.21-1.44)	9.1 (6.4-11.2)	2.3 (2-2.7)	3 (2.6-3.3)	0.94	27 (22-33)	0.52 (0.41-0.67)	0.98 (0.75-1.13)	0.64 (0.61-0.66)	25 (24.5-30.4)
E.corollae	0.83 (0.79-0.93)	5.2 (1.9-7.2)	1.3 (0.6-1.6)	2.3 (2.1-2.6)	0.99	64 (39-68)	0.19 (0.13-0.25)	0.36 (0.16-0.43)	0.61 (0.58-0.66)	12.7 (11.7-15)
S.torvus	1.37 (1.06-1.55)	4.6 (2.9-9.8)	1.1 (0.9-1.4)	3.8 (3.5-6.3)	0.91	15 (11-22)	0.2 (0.13-0.31)	0.35 (0.17-0.49)	0.69 (0.64-0.74)	23.9 (23.2-30.3)
S.ribesii	1.1 (0.89-1.13)	6.2 (3.3-6.7)	1.2 (0.9-1.4)	3 (2.8-3.6)	0.96	29 (21-41)	0.25 (0.18-0.36)	0.44 (0.26-0.57)	0.65 (0.58-0.68)	21 (18.5-26.6)
S.vitripennis	0.91 (0.89-1.02)	4.7 (4-6.8)	1.3 (0.9-1.5)	2.9 (2.5-3.2)	0.99	40 (36-51)	0.25 (0.19-0.28)	0.38 (0.35-0.47)	0.61 (0.58-0.66)	16.1 (13.4-18.2)
E.balteatus	0.77 (0.75-0.81)	4.9 (3-6)	1.3 (0.8-1.8)	2.6 (2.1-2.7)	0.47	43 (32-62)	0.18 (0.12-0.26)	0.41 (0.25-0.59)	0.58 (0.51-0.75)	20.9 (19.9-21.3)
M.cinctella	0.9 (0.76-1.12)	1.5 (0.8-4.5)	0.5 (0.3-0.8)	3.3 (2.6-4.1)	0.99	26 (22-51)	0.07 (0.05-0.12)	0.08 (0.05-0.28)	0.86 (0.77-0.9)	12.1 (10.7-15.1)
S.scripta	0.72 (0.66-0.76)	3.6 (1.5-5.1)	1 (0.6-1.3)	2.1 (2-2.5)	0.99	55 (40-66)	0.12 (0.09-0.21)	0.18 (0.13-0.32)	0.67 (0.6-0.87)	8.7 (7-10.1)
M.erraticus	0.97 (0.59-1.18)	5.2 (2.9-6.4)	1.3 (0.7-1.6)	2.8 (2.6-4)	0.96	24 (21-40)	0.24 (0.15-0.35)	0.55 (0.2-0.64)	0.63 (0.61-0.65)	23.4 (20.4-27.1)
H.trivittatus	1.51 (1.37-1.77)	3.7 (2.1-5.7)	1.1 (0.8-1.6)	3.5 (3.1-3.7)	0.98	24 (13-27)	0.28 (0.18-0.38)	0.34 (0.25-0.56)	0.7 (0.63-0.72)	26.8 (24.4-31.2)
H.pendulus	1.52 (1.45-1.56)	3.3 (2.6-3.6)	0.9 (0.8-1)	3.7 (3.2-4.2)	0.94	17 (14-27)	0.21 (0.14-0.25)	0.27 (0.22-0.39)	0.69 (0.67-0.75)	22.7 (21.6-23.3)
H.hybridus	1.61 (1.48-1.74)	5.2 (4.4-7.9)	1.3 (1.3-1.7)	3.2 (2.7-3.6)	0.97	31 (26-35)	0.33 (0.27-0.42)	0.58 (0.47-0.61)	0.7 (0.68-0.72)	28.1 (27.2-29.9)
E.sepulchralis	1.45 (1.42-1.56)	6.4 (4.2-8.1)	1.7 (1.5-2.4)	2.7 (2.5-2.9)	0.98	36 (33-38)	0.33 (0.23-0.41)	0.61 (0.45-0.7)	0.72 (0.59-0.75)	12.4 (11.7-13.1)
V.bombylans	2.41 (2.36-2.49)	4.3 (3.1-7.2)	1.8 (1.5-2.2)	4 (3.8-4)	0.95	14 (13-16)	0.42 (0.37-0.58)	0.73 (0.61-0.97)	0.78 (0.76-0.81)	43.7 (41.6-47.2)
V.pellucens	2.35 (2.13-2.67)	1.4 (1-2.1)	1.3 (1.2-1.5)	3.7 (3.7-4)	0.92	11 (11-15)	0.29 (0.27-0.32)	0.46 (0.39-0.49)	0.81 (0.75-0.86)	49.2 (47.5-55.1)
M.equestris	1.83 (1.7-1.95)	3.1 (2.9-5.7)	1.3 (1.1-1.5)	4.7 (4.6-5.5)	0.86	10 (9-13)	0.24 (0.21-0.27)	0.37 (0.33-0.43)	0.65 (0.63-0.66)	24.6 (23.1-25.2)
